# The West Palaearctic genera of Nematinae (Hymenoptera, Tenthredinidae)

**DOI:** 10.3897/zookeys.875.35748

**Published:** 2019-09-16

**Authors:** Marko Prous, Andrew Liston, Katja Kramp, Henri Savina, Hege Vårdal, Andreas Taeger

**Affiliations:** 1 Senckenberg Deutsches Entomologisches Institut, Eberswalder Str. 90, 15374 Müncheberg, Germany Senckenberg Deutsches Entomologisches Institut Müncheberg Germany; 2 Department of Zoology, Institute of Ecology and Earth Sciences, University of Tartu, Vanemuise 46, 51014 Tartu, Estonia University of Tartu Tartu Estonia; 3 Parc Majorelle, 33 chemin du Ramelet-Moundi, bât. C, apt. 16, 31100 Toulouse, France Unaffiliated Toulouse France; 4 Swedish Museum of Natural History, Box 50007, SE-10405 Stockholm, Sweden Swedish Museum of Natural History Stockholm Sweden

**Keywords:** Distribution, keys, lectotype designations, sawflies, Sweden, synonymy

## Abstract

Keys to adults and larvae of the genera of West Palaearctic nematine sawflies are presented. Species of some of the smaller genera are keyed, and their taxonomy, distribution, and host plants reviewed, with a geographic focus on north-western Europe, particularly Sweden. *Dinematus* Lacourt, 2006 is a new junior subjective synonym of *Pristiphora* Latreille, 1810, resulting in the new combination *Pristiphora krausi* (Lacourt, 2006) for the type species of *Dinematus*. *Hemichroa monticola* Ermolenko, 1960 is a new junior subjective synonym of *Hemichroa australis* (Serville, 1823). Lectotypes are designated for *Tenthredo opaca* Fabricius, 1775, Mesoneura opaca var. nigerrima Enslin, 1914, Mesoneura opaca var. obscuriventris Enslin, 1914, *Nematus hypogastricus* Hartig, 1837, *Nematus alnivorus* Hartig, 1840, *Leptopus rufipes* Förster, 1854, *Nematus protensus* Förster, 1854, and Platycampus luridiventris var. pleuritica Enslin, 1915. A phylogenetic analysis based on four genes (mitochondrial COI and nuclear NaK, POL2, and TPI) supports the current generic classification.

## Introduction

In 2012 a project funded by the Swedish Taxonomy Initiative was launched, with the main objective of improving our knowledge of the taxonomy and distribution of nematine sawflies in Fennoscandia, and Sweden in particular (STI Nematinae Group 2013). As a first step, the generic classification of the world Nematinae was revised by Prous et al. (2014), and the genera keyed. Here, we present a condensed version of that key, covering only the West Palaearctic genera, with which it should be possible to identify most specimens more easily. Included are treatments of the species of some smaller genera: *Hemichroa*, *Mesoneura*, *Neodineura*, *Platycampus*, and *Stauronematus*. The species of the other genera were either covered by Prous et al. (2017) and Liston et al. (2017, 2019a–c), or are to be dealt with in works currently in preparation. Geographic scope of the taxonomic treatments at genus / species group level varies between coverage of the whole West Palaearctic, to consideration only of the species which are known from Fennoscandia, or potentially present there. The differences in the size of regions covered for each genus / species group arise through the amount of material available for study, including fresh specimens suitable for genetic sequencing, and the perceived complexity of species-level taxonomy in the group. The present work thus represents an overview of all Nematinae known to occur in Fennoscandia, and in conjunction with the publications covering the remaining genera is intended to enable determination to species level of specimens of all nematine genera from north-west Europe.

## Materials and methods

The Swedish Malaise Trap Project is abbreviated to **SMTP**. Abbreviations for the names of collections referred to in the text are as follows:

**BMNH** Natural History Museum, London, United Kingdom

**FMNH** Finnish Museum of Natural History, Helsinki, Finland

**HNHM** Hungarian Natural History Museum, Budapest, Hungary

**LSUK** Linnean Society, London, United Kingdom

**MNHN** Muséum national d’Histoire naturelle, Paris, France

**MZFN** Museo Zoologico dell’Università Federico II, Naples, Italy

**MZLU** Lunds universitet, Entomology Collection, Lund, Sweden

**NFVG** Niedersächsische Forstliche Versuchsanstalt, Göttingen, Germany

**NHRS** Naturhistoriska riksmuseet, Stockholm, Sweden

**NMPC** National Museum (Natural History), Prague, Czech Republic

**RMNH** Naturalis Biodiversity Centre, Leiden, Netherlands

**SDEI** Senckenberg Deutsches Entomologisches Institut, Müncheberg, Germany

**TUZ** Natural History Museum, Tartu, Estonia

**ULQC** University of Laval, Quebec, Canada

**USNM** National Museum of Natural History, Washington D. C., USA

**ZMHB** Naturkundemuseum, Berlin, Germany

**ZMUC** Zoological Museum, University of Copenhagen, Copenhagen, Denmark

**ZSM**Zoologische Staatssammlung, Munich, Germany.

In the specimen data the dates are given as dd.mm.yyyy, and coordinates as positive (north or east) or negative (south or west) decimal degrees latitude and longitude.

Morphological terminology mostly follows Viitasaari (2002), but sawtooth is used instead of serrula (see Malagón-Aldana et al. 2017), and the large, ventrally situated, more or less triangular flange above each sawtooth is called a spurette (following Ross 1943; see Figs 108, 112 arrows). Images of complete imagines and morphological details were made at the SDEI with Leica cameras attached to a variety of microscopes. Composite images with an extended depth of field were created from stacks of images using the software CombineZP, and finally arranged and partly enhanced with Ulead PhotoImpact X3. Some of the figures were first published by Prous et al. (2014). Unless otherwise stated, photos of adults and larvae were made by AL, MP, HS, and AT.

First drafts of the key to larvae were based mainly on Lorenz and Kraus (1957), and subsequently modified to include the results of more recently published studies, and the examination of specimens available to us. The tree species known as Mountain Birch, which dominates large areas of vegetation in northern Fennoscandia, is referred to as Betula pubescens var. pumila (Zanoni ex Murray) Govaerts, following Plants of the World online (2017), which treats the formerly widely-used names *B. czerepanovii* N. I. Orlova and *B. tortuosa* Ledeb. as its synonyms.

DNA was extracted and purified with an EZNA Tissue DNA Kit (Omega Bio-tek) according to the manufacturer’s protocol and stored at -20 °C for later use. Typically, one or two legs were used for DNA extraction, but for males the whole genital capsule was often additionally used to increase DNA yield and to free penis valves from muscles before photography. In some cases, the whole specimen was used for extraction. One mitochondrial and four nuclear regions were used in the phylogenetic analyses, although not all of these genes were obtained for all species. Primers used for amplification and sequencing are listed in Table 1. The mitochondrial region used is a large fragment (1078–1087 bp depending on the primer set) of the cytochrome oxidase subunit I gene (COI). The fragment includes the entire standard barcode region (658 bp) of the animal kingdom (Hebert et al. 2003). The nuclear markers used are fragments of sodium/potassium-transporting ATPase subunit alpha (NaK), triose-phosphate isomerase (TPI), DNA dependent RNA polymerase II subunit RPB1 (POL2), and transformation/transcription domain-associated protein (TRRAP). The NaK fragment used is a nearly complete sequence of its longest exon, 1654 bp. The TPI fragment used is the nearly complete gene region, containing 676 bp of three exons and two short introns (each around 50–100 bp) in Nematinae, altogether 788–842 bp. The POL2 fragment used is composed of two partial exons (together 2407–2623 bp depending on the primer set) and one short intron (67–86 bp). The TRRAP fragment used is a 3379 bp fragment of its longest exon (sequenced only for *Hoplocampa* and *Monocellicampa*). New POL2 and TRRAP primers were designed mainly based on four sawfly genomes (accessions AOFN02000108, AOFN02000124 [*Athalia rosae*], LGIB01000723, LGIB01000528 [*Neodiprion lecontei*], AMWH01002735, AMWH01006798 [*Cephus cinctus*], AZGP02002036, AZGP02002013 [*Orussus abietinus*]) and transcriptomes (Misof et al. 2014, Peters et al. 2017) available in GenBank. Numbers in the new POL2 and TRRAP primer names refer to the binding position of the 3’ end of each primer in the coding region of *Athalia rosae* mRNA (accessions XM_012395805 and XM_012406083).

**Table 1.** Primers used for PCR and sequencing (preferred primers in bold), with information provided on respective gene fragment, primer name, direction (forward, F or reverse, R), primer sequence, standard PCR annealing temperature, utilization (PCR/ sequencing), and reference. Primer annealing temperatures used for sequencing at Macrogen were usually 50 °C (47–50 °C).

Gene region	Primer name	F/R	Primer sequence 5'–3'	PCR annealing temperature (°C)	PCR/ Sequencing	Reference
COI	SymF1	F	TTTCAACWAATCATAAARAYATTGG	49	PCR, seq	(Prous et al. 2016)
COI	**SymF4**	F	AAATGATTATTYTCWACWAATCAYAA	50	PCR, seq	This study
COI	**sym-C1-J1718**	F	GGAGGATTTGGAAAYTGAYTAGTWCC	49	PCR, seq	(Nyman et al. 2006)
COI	**symC1-J1751**	F	GGAGCNCCTGATATAGCWTTYCC	47	seq	(Prous et al. 2016)
COI	**SymR1**	R	TAAACTTCWGGRTGICCAAARAATC	47	PCR, seq	(Prous et al. 2016)
COI	SymR2	R	TAAACTTCTGGRTGTCCAAARAATCA	47	PCR, seq	(Prous et al. 2016)
COI	**A2590**	R	GCTCCTATTGATARWACATARTGRAAATG	49	PCR, seq	(Normark et al. 1999)
NaK	**NaK_263F**	F	CTYAGCCAYGCRAARGCRAARGA	59	PCR, seq	(Prous et al. 2017)
NaK	**NaK_809F**	F	GCWTTYTTCTCNACSAAYGCSGTNGARGG	55	PCR, seq	(Prous et al. 2017)
NaK	**NaK_907Ri**	R	TGRATRAARTGRTGRATYTCYTTIGC	54	PCR, seq	(Prous et al. 2017)
NaK	NaK_910R	R	TGRATRAARTGRTGRATYTCYTT	50	PCR, seq	(Prous et al. 2017)
NaK	NaK_1250Fi	F	ATGTGGTTYGAYAAYCARATYATIGA	56	PCR, seq	(Prous et al. 2017)
NaK	**NaK_1250Fv2**	F	ATGTGGTTYGAYAAYCARATHATIGA	56	PCR, seq	This study
NaK	**NaKRev475**	R	TCGATRATYTGRTTRTCRAACCACAT	56	seq	(Leppänen et al. 2012)
NaK	**NaK_1498R**	R	ACYTGRTAYTTGTTNGTNGARTTRAA	52	PCR, seq	(Prous et al. 2019)
NaK	**NaK_1918R**	R	GATTTGGCAATNGCTTTGGCAGTDAT	59	PCR, seq	(Prous et al. 2017)
POL2	POL2_104Fi	F	GYATGTCAGTYACNGATGGIGG	59	PCR, seq	(Prous et al. 2019)
POL2	**POL2_104Fv2**	F	CGNATGTCNGTNACNGAYGGIGG	60	PCR, seq	(Prous et al. 2019)
POL2	**POL2_574R**	R	TCYTCRTTNACRTGYTTCCAYTCNGC	59	seq	(Prous et al. 2019)
POL2	POL2_599F	F	GARTGGAARCAYGTVAAYGARGA	54	PCR, seq	(Prous et al. 2019)
POL2	**POL2_797F**	F	ATGTAYGGNTCNGCNAARAAYCARGA	58	PCR, seq	(Prous et al. 2019)
POL2	POL2_889R	R	TGRAAYTGYARCATYTTWATRTTYTC	52	PCR, seq	(Prous et al. 2019)
POL2	**POL2_928R**	R	GGCATNCCNGGCATRTCRTTRTCNAC	59	PCR, seq	(Prous et al. 2019)
POL2	**POL2_1388F**	F	CAYAARATGAGTATGATGGG	51	PCR, seq	(Prous et al. 2019)
POL2	POL2_1459R	R	TTCATYTCRTCNCCRTCRAARTC	52	PCR, seq	(Prous et al. 2019)
POL2	**POL2_1706F**	F	TGGGAYGGNAARATGCCNCARCC	60	PCR, seq	(Prous et al. 2019)
POL2	**POL2_1732R**	R	GARAADATYTGYTTNCCNGTCCA	55	PCR, seq	This study
POL2	POL2_1759R	R	ATCATRTTNACRTTNCCNGGDATDAT	55	PCR, seq	(Prous et al. 2019)
POL2	POL2_1777Ri	R	GTRCTGTGIGTYCKDATCATRTT	55	PCR, seq	(Prous et al. 2019)
POL2	POL2 hym 3F	F	ACNCACAGYACNCAYCCN GAYGA	56	seq	(Malm and Nyman 2015)
POL2	POL2_2423F	F	CATTTYATHAARGAYGAYTAYGG	51	seq	(Prous et al. 2019)
POL2	POL2_2509R	R	TTNACRGCRGTATCRATNAGACCYTC	60	PCR, seq	(Prous et al. 2019)
POL2	**POL2_2569R**	R	TGNACCATNACNGAYTCCATAGCYTTDAT	60	PCR, seq	This study
POL2	POL2_2725R	R	GGATCRAAYTTRAAYTTYTTYTC	50	PCR, seq	(Prous et al. 2019)
TPI	**TPI_29Fi**	F	GYAAATTYTTYGTTGGNGGIAA	52	PCR, seq	(Prous et al. 2016)
TPI	**TPI385Fi**	F	GTRATYGCNTGYATYGGIGARA	52	seq	(Prous et al. 2016)
TPI	**TPI 275Ri**	R	GCCCANACNGGYTCRTAIGC	56	seq	(Malm and Nyman 2015)
TPI	**TPI706R**	R	ACNATYTGTACRAARTCWGGYTT	52	PCR, seq	(Prous et al. 2016)
TRRAP	**TRRAP_833F**	F	AAYAARGARGTNTTYGTNGAYTTYATGGG	58	PCR, seq	This study
TRRAP	**TRRAP_1658F**	F	CARTCNAARCARTTYCARCCNAARGARAC	60	seq	This study
TRRAP	TRRAP_1702R	R	GGNGGNCCDATNGTRTARATRTC	56	seq	This study
TRRAP	**TRRAP_1831R**	R	AADATYTCYTGRAANGTYTGNGGRTTCAT	59	seq	This study
TRRAP	**TRRAP_2648Fi**	F	ATGATGATHGARCCNCARAARYTNGAITA	58	PCR, seq	This study
TRRAP	**TRRAP_3046R**	R	TGNGCDATNGCNACCATNGTRTARTG	60	PCR, seq	This study
TRRAP	**TRRAP_3482Fi**	F	GTNTCNAAYGGNGCHATHGAYATGGCIAA	62	seq	This study
TRRAP	**TRRAP_3685Ri**	R	ACYTCYTTRTGNGGYTCCATNACYTCIGT	62	PCR, seq	This study
TRRAP	TRRAP_4086F	F	CARGARGCNGCNTTYGARTGYATG	59	seq	This study
TRRAP	**TRRAP_4213Ri**	R	CTRAANGTRCTNGGRAANARYTGIGT	56	PCR, seq	This study

PCR reactions were carried out in a total volume of 15–35 μl containing 1.0–2.5 μl of extracted DNA, 1.5–3.5 μl (5.0–15 pmol) of primers and 7.5–17.5 μl of 2× Multiplex PCR Plus Master mix (QIAGEN). The PCR protocol consisted of an initial DNA polymerase (HotStar Taq) activation step at 95 °C for 5 min, followed by 38–40 cycles of 30 s at 95 °C, 90–120 s at 49–60 °C (depending on the primer set used), and 70–180 s (depending on the amplicon size) at 72 °C; the last cycle was followed by a final 30 min extension step at 68 °C. COI (primers symF4 [or symF1] + A2590), NaK (NaK_263F + 1918R) and TPI (TPI_29Fi + TPI706R) were in most cases amplified in one fragment, POL2 in one to three fragments, and TRRAP in two fragments (TRRAP_833F + 3046R and TRRAP_2648Fi + 4213Ri). Three μl of PCR product was visualised on a 1.4% agarose gel and the remaining product was then purified with FastAP and Exonuclease I (Thermo Scientific). 1.0–2.2 U of both enzymes were added to 12–32 μl of PCR solution and incubated for 15 min at 37 °C, followed by 15 min at 85 °C. 2–5 μl of purified PCR product per primer in a total volume of 10 μl (5–8 μl of sequencing primer at concentration 5 pmol/μl) were sent to Macrogen Europe (Netherlands) for sequencing. Both sense and antisense strands were sequenced using the primers listed in Table 1. Ambiguous positions (i.e., double peaks in chromatograms of both strands) due to heterozygosity were coded using IUPAC symbols. Sequences reported here have been deposited in the GenBank (NCBI) database (accession numbers MK624656–MK624923 and MK720818–MK720821), although not all of them are analysed here (covered in further publications on some of the genera not treated here). Some of the sequences analysed here were originally published by Schmidt et al. (2017) and Prous et al. (2016, 2017). Alignment of COI, NaK, and TRRAP sequences was straightforward because of the lack of indels (insertions or deletions). Alignment of POL2 and TPI was also straightforward without introns, but these were retained in some analyses published elsewhere (Liston et al. 2019a) and aligned manually. To concatenate separate gene alignments, we used R (R Core Team 2018) package *apex* (Jombart et al. 2017). For phylogenetic analyses we used the maximum likelihood method (ML) implemented in IQ-TREE 1.5.6 (http://www.iqtree.org/) (Nguyen et al. 2015). By default, IQ-TREE runs ModelFinder (Kalyaanamoorthy et al. 2017) to find the best-fit substitution model and then reconstructs the tree using the model selected according to Bayesian information criterion (BIC). We complemented this default option with SH-like approximate likelihood ratio (SH-aLRT) test (Guindon et al. 2010) and ultrafast bootstrap (Hoang et al. 2017) with 1000 replicates to estimate robustness of reconstructed splits. Minimal p-distances between and maximal distances within BIN (Barcode Index Number) clusters were taken from BOLD (http://www.boldsystems.org/) BIN database. Some of the COI barcode sequences used here were obtained from BOLD (http://www.boldsystems.org/). In this case, DNA extraction, PCR amplification, and sequencing were conducted at the Canadian Centre for DNA Barcoding (CCDB) in Guelph, Canada, using standardised high-throughput protocols (Ivanova et al. 2006, deWaard et al. 2008), available online under www.ccdb.ca/resources.php. DNA aliquots of SDEI vouchers are deposited in the DNA storage facility of the SDEI (including those that were originally extracted in CCDB).

## Results

**Figure 1.**
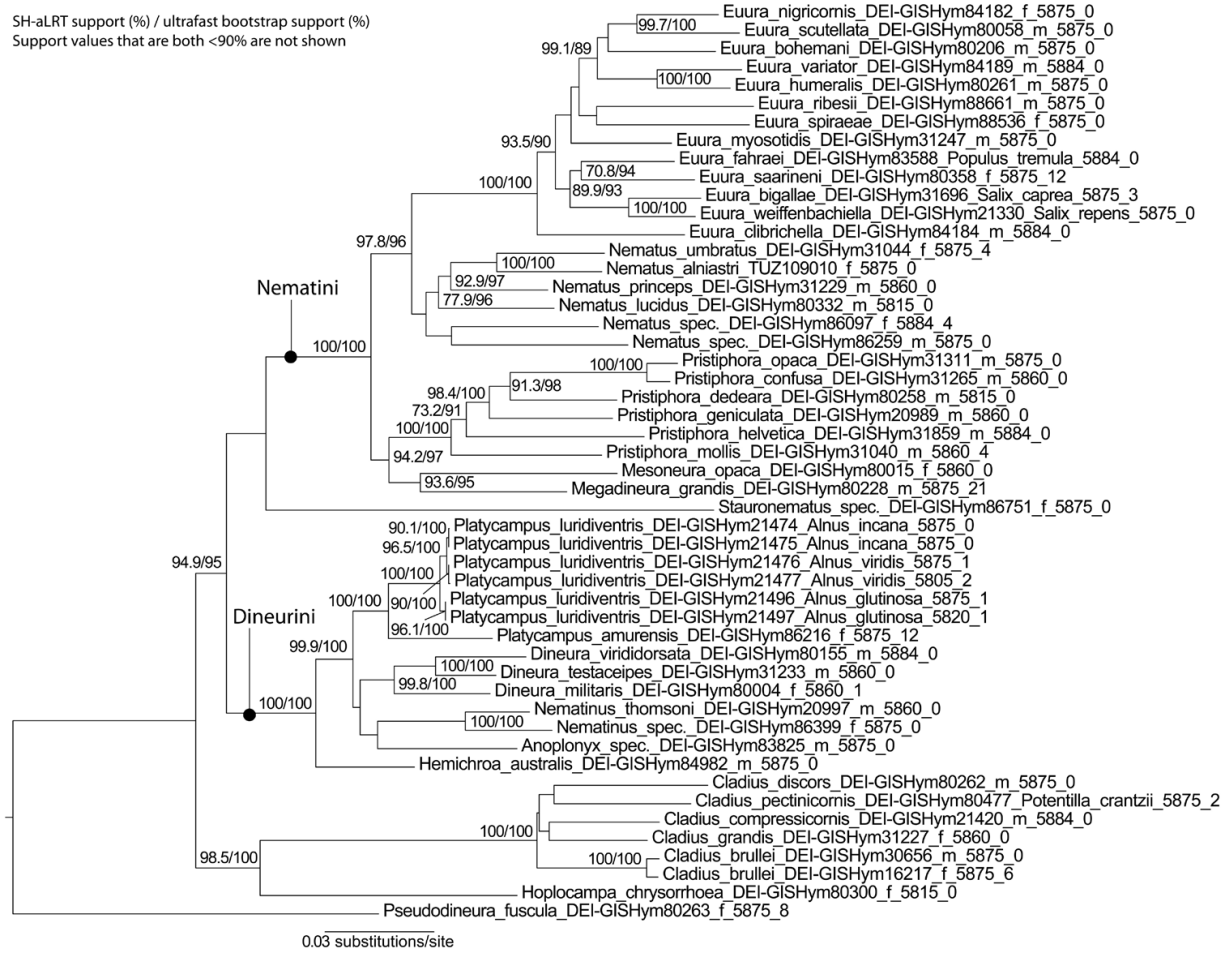
Maximum likelihood tree of Nematinae based on four genes (COI, NaK, POL2, TPI). Only specimens sequenced for all four genes were included. Short introns from POL2 and TPI were excluded. The best-fit model chosen according to Bayesian information criterion was GTR+R4. Numbers at branches show SH-aLRT support (%) / ultrafast bootstrap support (%) values. Support values for weakly supported branches (<90) are not shown. Letters “f” and “m” stand for “female” and “male”, and are not given for larvae. Numbers at the end of the tip labels refer to the length of the sequence and the number of ambiguous positions (e.g., heterozygosities). The number of ambiguous positions given for two males are due to variation in mitochondrial COI because of possible heteroplasmy. The tree was rooted as in Prous et al. (2014). The scale bar shows the number of estimated substitutions per nucleotide position.

Previous taxonomic publications have mostly recognised several tribes within the Nematinae. For example, Vikberg (1982) allocated the North European genera to six tribes, of which his Nematini was further divided into three sub-tribes. Subsequently, additional tribes were erected, often for species-poor lineages with more or less distinctive morphological and biological characters, e.g., Pristicampini (Zinovjev 1993), Stauronematini, and Bacconematini (Lacourt 1998). The circumscription of the tribes, and even of the Nematinae itself, has varied considerably between authors. Lacourt (1998), for example, removed *Cladius*, *Hoplocampa*, and *Susana* from the Nematinae, and treated each of these as a separate subfamily of Tenthredinidae. A clearer and more objective assessment of suprageneric classification was first achieved with the application of genetic data by Nyman et al. (2006). A second analysis in Prous et al. (2014), based on extended taxon sampling and more genes, yielded essentially similar results. A further refinement based on mitochondrial COI and three nuclear genes (NaK, POL2, TPI), with stronger support for some clades, is presented in Fig. 1. Noteworthy is that Nyman et al. (2006), Prous et al. (2014), and Malm and Nyman (2015) all recovered the Nematinae as monophyletic and indicated that *Cladius* (missing in Malm and Nyman 2015), *Hoplocampa*, and *Susana* do belong to the subfamily. Because monophyly of Nematinae is unambiguously supported based on previous analyses using the same genes, we did not test this here further. Our analyses of the subfamily without outgroups supports the previous generic classification as proposed in Prous et al. (2014). Because of limited sampling, Prous et al. (2014) were unable to state whether the three subgenera of *Cladius* are monophyletic, but based on expanded sampling, we now find that the largest subgenus Priophorus is not (Fig. 1). Because the delimitation of the subgenera of *Cladius* is problematic also morphologically, we propose here to abandon subgeneric classification until better evidence justifies it. Whether the various tribal names which have been proposed for single genera have much practical value is questionable. *Hoplocampa*, *Stauronematus*, and *Susana*, for example, although apparently phylogenetically isolated from other genera, are more clearly referred to by using their generic names. This will remain so at least until genetic data become available for a number of morphologically distinctive genus-series taxa. In the West Palaearctic, genetic data are still lacking for *Armenocampus*, *Neodineura*, and *Nescianeura*. On the other hand, to simplify discussions on phylogeny and biodiversity, use of the tribal names Nematini (equivalent to the “higher Nematinae” of Prous et al. 2014), Dineurini, and Pseudodineurini seems justified and useful. Support for Nematini and Dineurini (Pseudodineurini could not be tested because of the lack of sampling) in our molecular phylogeny is unambiguous (Fig. 1). Formally, the West Palaearctic genera belong to the following tribes:

Dineurini: *Anoplonyx*, *Dineura*, *Hemichroa*, *Nematinus*, *Platycampus* [and *Neodineura*?]

Nematini: *Euura*, *Mesoneura*, *Nematus*, *Pristiphora* [and *Nescianeura*?]

Pseudodineurini: *Endophytus*, *Pseudodineura*

Cladiini: *Cladius*

Hoplocampini: *Hoplocampa*

Stauronematini: *Stauronematus*

### Key to the West Palaearctic genera and selected species of Nematinae (imagines)

Genera and species represented in Fennoscandia are marked with an asterisk (*). Species numbers are for the West Palaearctic realm, followed by Fennoscandia.

**Table:** 

1	**a** Fore wing normal, veins normally developed (Figs 2–3)	**12**
–	**aa** Fore wing shortened, apex usually not reaching to the tip of the abdomen, veins often strongly aberrant (Figs 4–5) [some females of one arctic-alpine species]	* ***Euura abnormis* (Holmgren, 1883)** ♀
2(1)	**a** Vein 2A of hind wing complete, cell A closed (Fig. 5); **b** Body length 2–12 mm; **c** Vein 2r-rs frequently absent (Fig. 8) (ca. 600 species)	**3**
–	**aa** Vein 2A of hind wing incomplete, cell A open distally (Fig. 6); **bb** Body length 2–6 mm; **cc** Vein 2r-rs usually present (compare Fig. 9) (7 species)	**12**
3(2)	**a** Vein 2r-rs absent (Fig. 8) (more than 550 species)	**4**
–	**aa** Vein 2r-rs present (Fig. 9) (less than 30 species)	**13**
4(3)	**a** Base of vein 2A+3A incomplete and straight, cell PA open distally (Fig. 10) (more than 500 species)	**5**
–	**aa** Base of vein 2A+3A complete and curved up to 1A, cell PA closed (Fig. 11) (ca. 25 / 15* species)	**9**
5(4,18)	**a** Apex of vein C of fore wing swollen; at the point of origin of vein Rs+M from R, cell c usually only approx. as wide as R (Fig. 12); **b** Clypeus more or less truncate, at most slightly emarginate (Fig. 14); **c** Claws usually with subapical tooth (cf. Figs 18, 19), sometimes bifid or simple (Fig. 17), but never with basal lobe; **d** Valvula 3 frequently distinctly emarginate apically in dorsal view (Fig. 21); **e** Tangium of lancet with campaniform sensilla (“pores”) (Fig. 25), rarely absent (see Prous et al. 2017); **f** Tergum 8 in males of most species without distinct apical projection (Fig. 23), see Prous et al. (2017); **g** Valvispina of penis valve in many species at ventral margin (Fig. 27; see also Prous et al. 2017) (ca. 120 / 90* species)	* ***Pristiphora* Latreille, 1810**
–	**aa** Apex of vein C of fore wing often less swollen; at the point of origin of vein Rs+M from R, cell c approx. twice as wide as R or wider (Fig. 13); **bb** Clypeus usually at least one third deep emarginate (Fig. 15); exceptionally, truncate; **cc** Claws of various shape, but frequently bifid (cf. Fig. 20), rarely with basal lobe (Fig. 16); **dd** Valvula 3 only exceptionally emarginate apically in dorsal view (Fig. 22); **ee** Tangium of lancet without campaniform sensilla (Fig. 26); **ff** Tergum 8 in males often with distinct apical projection (Fig. 24); **gg** Valvispina of penis valve often distinctly removed from ventral margin (Fig. 28)	**6**
6(5)	**a** Claws with basal lobe in addition to subapical tooth, subapical tooth erect and well separated from apical tooth, longer than apical tooth (Fig. 16); **b** Clypeus more or less truncate (2 / 1* species)	* ***Stauronematus* Benson, 1953**
–	**aa** Claws without basal lobe (Figs 17–20), subapical tooth usually shorter than apical tooth (Figs 18–19), sometimes claws simple (Fig. 17); **bb** Clypeus usually at least emarginate to one third depth; exceptionally, truncate	**7**
7(6)	**a** Vein Sc before point of origin of vein M from R (Fig. 29) (most species)	**8**
–	**aa** Vein Sc beyond point of origin of vein M from R (Fig. 30) (few species)	**16**
8(7)	**a** In female, abdominal tergum 9 in lateral view more than 3 times as long as tergum 8 (Fig. 31); **b** In male, pseudoceps apically strongly narrowed, often forming distinct filament (Figs 33–34, figs 7–11 in Lindqvist 1957, http://doi.org/10.6084/m9.figshare.5100877); **c** Left mandible in lateral view tapered evenly towards apex (Figs 36–37) (8 / 7* species)	* ***Nematinus* Rohwer, 1911**
–	**aa** In female, abdominal tergum 9 in lateral view usually less than 2 times as long as tergum 8 (Fig. 32); **bb** In male, penis valve without distinct filament (Fig. 35); **cc** Left mandible in lateral view usually markedly constricted near middle (Fig. 38). Two genera which are currently only separated genetically, not morphologically; exceptionally, specimens of *Pristiphora* might also run here (ca. 440 / *number of Fennoscandian species still unclear)	*Euura Newman, 1837 and (13 /10* species) *Nematus Panzer, 1801

**Figures 2–13.**
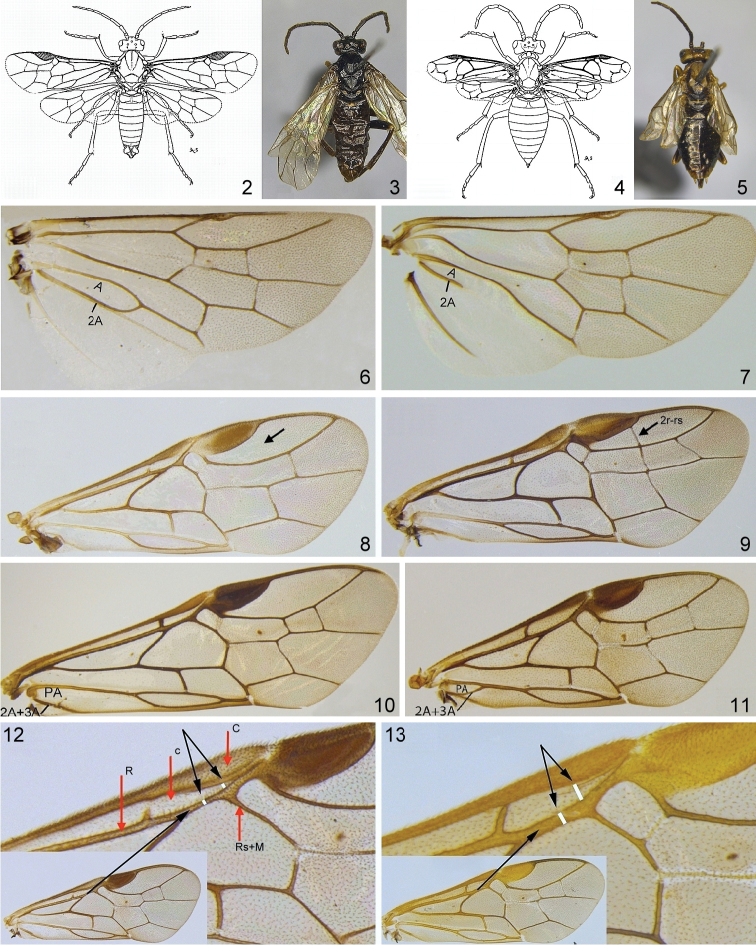
Generic characters of Nematinae**2–3***Euura abnormis* ♂ **4, 5***Euura abnormis* ♀ (drawings after Benson 1958) **6***Hoplocampa chrysorrhoea* rear wing **7***Pseudodineura enslini* rear wing **8***Euura mucronata* fore wing **9***Mesoneura opaca* fore wing **10***Nematus lucidus* fore wing **11***Platycampus luridiventris* fore wing **12***Pristiphora pallidiventris* fore wing **13***Euura annulata* fore wing.

**Figures 14–28.**
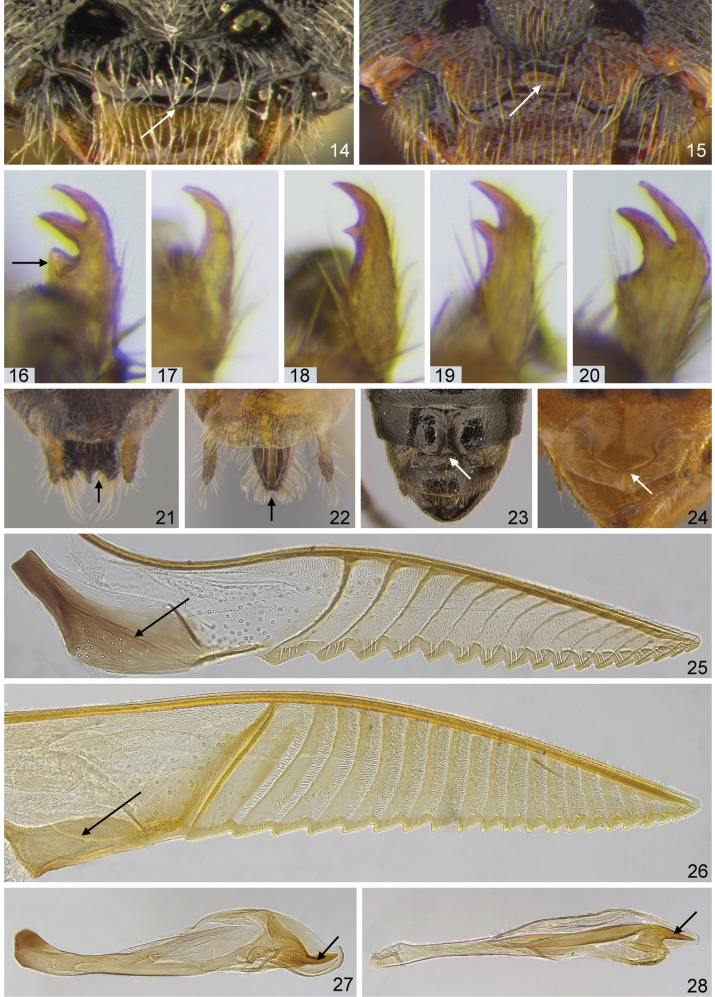
Generic characters of Nematinae**14***Pristiphora dedeara* clypeus **15***Nematus septentrionalis* clypeus **16***Stauronematus platycerus* claw (arrow: basal lobe) **17***Euura pumilio* claw **18***E. clitellata* claw **19***Nematus lucidus* claw **20***E. ribesii* claw **21***Pristiphora pallidiventris* valvula 3 (arrow: emargination) **22***Euura reticulata* valvula 3 (arrow: not emarginate) **23***Pristiphora subopaca* tergum 8 **24***Euura ribesii***25***Pristiphora astragali* lancet (arrow: campaniform sensilla on tangium) **26***Euura bertilpoppii* lancet (arrow: no campaniform sensilla on tangium) **27***Pristiphora pseudodecipiens* penis valve (arrow: valvispina) **28***Euura jugicola* penis valve (arrow: valvispina).

**Figures 29–40.**
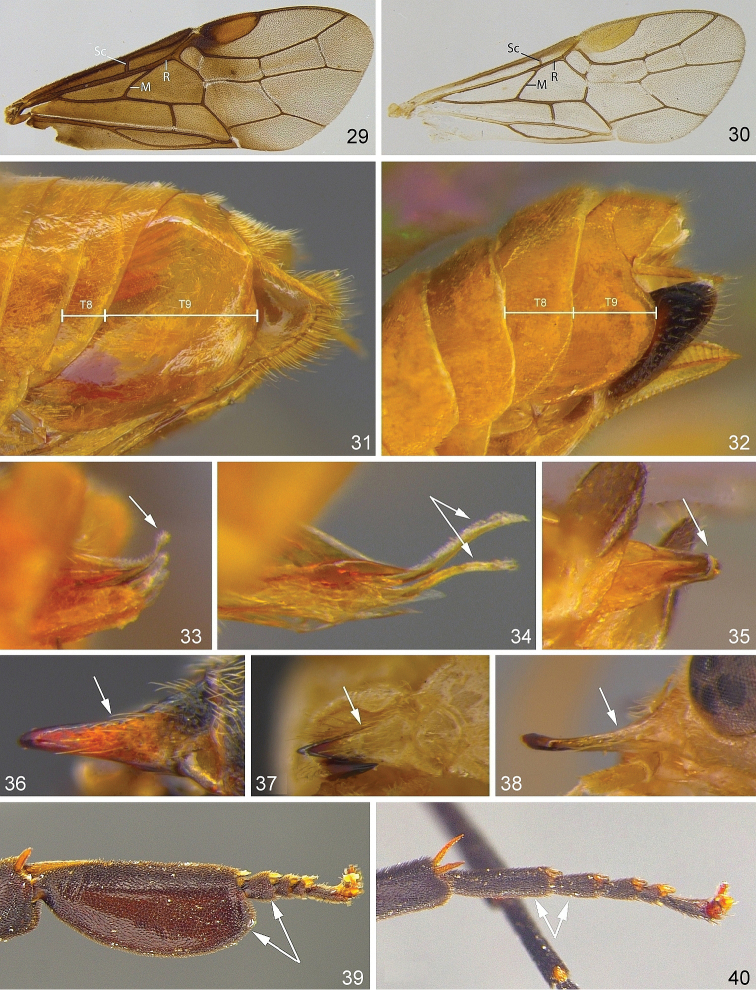
Generic characters of Nematinae**29***Nematinus fuscipennis* fore wing **30***Dineura virididorsata* fore wing **31***Nematinus fuscipennis* abdomen tip **32***Euura vesicator* abdomen tip **33***Nematinus fuscipennis* penis valve **34***Nematinus bilineatus* penis valve **35***Euura vesicator* penis valve **36***Nematinus fuscipennis* left mandible **37***Dineura virididorsata* left mandible **38***Pristiphora krausi* left mandible **39***Nematus septentrionalis* metatarsus **40***Euura caeruleocarpus* metatarsus.

Preliminarily, the European *Nematus* species may be separated morphologically from *Euura* as follows:

**Table:** 

A	(**a**) 1^st^ metatarsomere 2.0–3.0 times as wide as width of 2^nd^ metatarsomere (Fig. 39) (formerly *Craesus*) (6 / 3* species)	* ***Nematus septentrionalis* group**
–	(**b**) 1^st^ metatarsomere only slightly wider than width of 2^nd^ metatarsomere (Fig. 40)	**B**
B(A)	(**a**) Pterostigma dark brown to black (Figs 41–43, 56–58); (**b**) Antennae black (Figs 41–43); (**c**) Pronotal angles and tegulae reddish or yellowish (Figs 41–43)	**C**
–	(**aa**)–(**cc**) Characters not in the combination of (**a**)–(**c**): (**aa**) Pterostigma often mainly pale; (**bb**) Antennae frequently (especially ventrally) pale; (**cc**) Pronotal angles and / or tegulae may be black	**F**
C(B)	(**a**) Mesepisternum densely sculptured, ± matt; (**b**) Terga (1–)2–3(–6), femora, tibiae, and tarsi of fore and middle legs reddish (Figs 41–42); (**c**) Body 7–11 mm, torpedo-shaped (Figs 41–42)	* ***Nematus lucidus* (Panzer, 1801)**
–	(**aa**) Mesepisternum shiny, at most weakly sculptured; (**bb**) Coloration different (Figs 43, 56–58); (**cc**) Body 5–10.5 mm, usually not torpedo-shaped	**D**
D(C)	(**a**) Abdomen black (Fig. 43); (**b**) Thorax black (except for tegulae and pronotum); (**c**) Legs largely pale (hind tibia with basal half pale, apical half black or reddish with black apex) (Fig. 43); (**d**) Valvula 3 in dorsal view narrowing towards the apex, apically broadly rounded (Fig. 44); (**e**) Paravalva of penis valve roughly oval-shaped and distinctly longer than valvura, valvispina distinctly removed from ventral margin and paravalva with a small lobe at base of valvispina (Fig. 50). Larva on *Lonicera* (formerly *Paranematus*). (5 / 5* species)	* ***Nematus wahlbergi* group**
–	(**aa**) Abdomen usually at least partly yellowish or reddish (Fig. 56); (**bb**) Thorax often at least laterally ± yellowish (Fig. 56); (**cc**)–(**ee**) Characters often different	**E**
E(D)	(**a**) Valvula 3 in dorsal view hardly tapering towards apex, and visible parts approx. as long as broad (Fig. 45); bases of longest setae on each valvula nearly parallel (Fig. 45); (**b**) Straight and gradually narrowing valvispina of penis valve roughly in the middle of paravalva, paravalva excluding valvispina distinctly shorter than pseudoceps, ventroapical lobe of paravalva extending ca. 1/3 of length of valvispina, basal third or half of valvar strut more or less at the ventral margin of paravalva (Fig. 51)	* ***Nematus umbratus* Thomson, 1871**
–	(**aa**) Valvula 3 in dorsal view tapering towards apex, and visible parts *often* longer than broad (Fig. 48); bases of longest setae on each valvula 3 *often* strongly divergent from each other (Figs 46–47, 49); (**bb**) Penis valve different (Figs 52–54)	*Euura* part. (*melanocephalus, *bohemani, *ribesii species group, *salicis)
F(B)	(**a**) Pronotal angles black (Figs 57–58); (**b**) Body 8–12 mm, torpedo-shaped (Fig. 57); (**c**) Abdomen black with 3^rd^ and 4^th^ segment ± pale (alive: green) (Fig. 58) or sometimes completely black in males; (**d**) Valvispina of penis valve roughly in the middle of paravalva and with a distinct hook; dorsal part of anterior margin of paravalva at base of valvispina more basal than ventral part, but both margins roughly perpendicular to valvispina; basal third of valvar strut more or less at the ventral margin of paravalva (Fig. 55)	* ***Nematus princeps* Zaddach, 1876**
–	(**aa**) Pronotal angles often pale marked; (**bb**) Body length frequently less than 8 mm, usually not torpedo-shaped; (**cc**) Abdomen coloured differently (**dd**) Penis valve different	***Euura* part**
9(4)	**a** Vein 2m-cu running into cell 2Rs (Fig. 59) (in few aberrant specimens into cell 1Rs, very slightly distal to 2r-m, or vein 2r-m absent); **b** Length of vein R in the fore wing between junctions with veins M and Rs+M usually not longer than first sector of Rs (Fig. 59	**10**
–	**aa** Vein 2m-cu running into cell 1Rs (Fig. 60); **bb** Length of vein R in the fore wing between junctions with veins M and Rs+M clearly longer than first sector of Rs (Fig. 60)	**11**
10(9)	**a** Claw usually with large or small inner tooth; exceptionally, simple; **b** Scape and pedicellus together much shorter than the first flagellomere, sometimes in male the latter with basal projection (Fig. 61) (11 / 8* species)	* ***Cladius* Illiger, 1807**
–	**aa** Claw simple; **bb** Scape and pedicellus together approx. as long as the first flagellomere, the latter without projection (Fig. 62) (Only one rare species from Armenia, *A. necopinus* (Zhelochovtsev, 1941); not examined)	[***Armenocampus* Zinovjev, 2000**]
11(9)	**a** Claw simple, without subapical tooth; **b** Apex of vein C of fore wing swollen; at the point of origin of vein Rs+M from R, cell c usually only approx. as wide as R (cf. Fig. 65) (5 / 4* species)	* ***Anoplonyx* Marlatt, 1896**
–	**aa** Claw with subapical tooth; **bb** Apex of vein C of fore wing less swollen; at the point of origin of vein Rs+M from R, cell c approx. twice as wide as R or wider (cf. Fig. 66) (2? /1* species)	* ***Platycampus* Schiödte, 1839**
12(2)	**a** Base of vein 2A+3A incomplete and straight (Fig. 63); **b** Vein 2r-m usually present (Fig. 63); **c** Vein 2m-cu present (Fig. 63) (6/ 3* species; see key in Liston et al. 2019b)	* ***Pseudodineura* Konow, 1885**
–	**aa** Base of vein 2A+3A more or less complete and curved up to 1A (Fig. 64); **bb** Vein 2r-m of fore wing often absent (Fig. 64); **cc** Vein 2m-cu absent or present (Only *E. anemones* (Hering, 1924)*)	*Endophytus Hering, 1934
13(3)	**a** Base of vein 2A+3A complete and curved up to 1A (Fig. 64)	**14**
–	**aa** Base of vein 2A+3A incomplete and straight (Fig. 63)	**15**
14(13)	**a** Vein 2m-cu running into cell 2Rs (Fig. 65); **b** Apex of vein C of fore wing swollen; at the point of origin of vein Rs+M from R, cell c usually only approx. as wide as R (in pale specimens may be hardly visible) (Fig. 65); **c** Body length 3–7 mm, frequently less than 5 mm (14 / 9* species; see key in Liston et al. 2019c)	*Hoplocampa Hartig, 1837
–	**aa** Vein 2m-cu running into cell 1Rs (Fig. 66); **bb** Apex of vein C of fore wing less swollen; at the point of origin of vein Rs+M from R, cell c approx. twice as wide as R or wider (Fig. 66); **cc** Body length 5–8 mm (2 / 2* species)	*Hemichroa Stephens, 1835
15(13)	**a** Vein Sc before point of origin of vein M from R (cf. Fig. 29)	**17**
–	**aa** Vein Sc beyond point of origin of vein M from R (Fig. 30)	*Dineura Dahlbom, 1835
16(7)	**a** Left mandible in lateral view markedly constricted near middle (cf. Fig. 38); **b** Head, legs, thorax ventrally, valvifer 2 and valvula 3 black; abdomen and mesonotum yellow or orange (Figs 123–126) (one very rare species: *N. noblecourti* Lacourt, 2006)	***Nescianeura* Lacourt, 2006**
–	**aa** Left mandible in lateral view tapered regularly towards apex (Figs 36–37); **bb** Coloured differently (4 / 4* species; see key in Liston et al. 2019a).	*Dineura Dahlbom, 1835
17(15)	**a** Clypeus long (Fig. 67); **b** Labrum short, apically emarginate (Fig. 67); **c** Left mandible in lateral view tapered regularly towards apex (Figs 36–37) (One very rare species: *N. arquata* (Klug, 1816))	***Neodineura* Taeger, 1989**
–	**aa** Clypeus short (Fig. 68); **bb** Labrum normal, apically rounded (Fig. 68); **cc** Left mandible in lateral view markedly constricted near middle (cf. Fig. 38)	**18**
18(17)	**a** Antenna rather short, ca. 1.5 times as long as width of head; **b** Claw with large inner tooth (2 / 1* species)	*Mesoneura Hartig, 1837
–	**aa** Antenna longer, ca. 2–3 times as long as width of head; **bb** Claw simple or with small inner tooth (few specimens of *Pristiphora*; see key in Prous et al. 2017)	**5**

**Figures 41–58.**
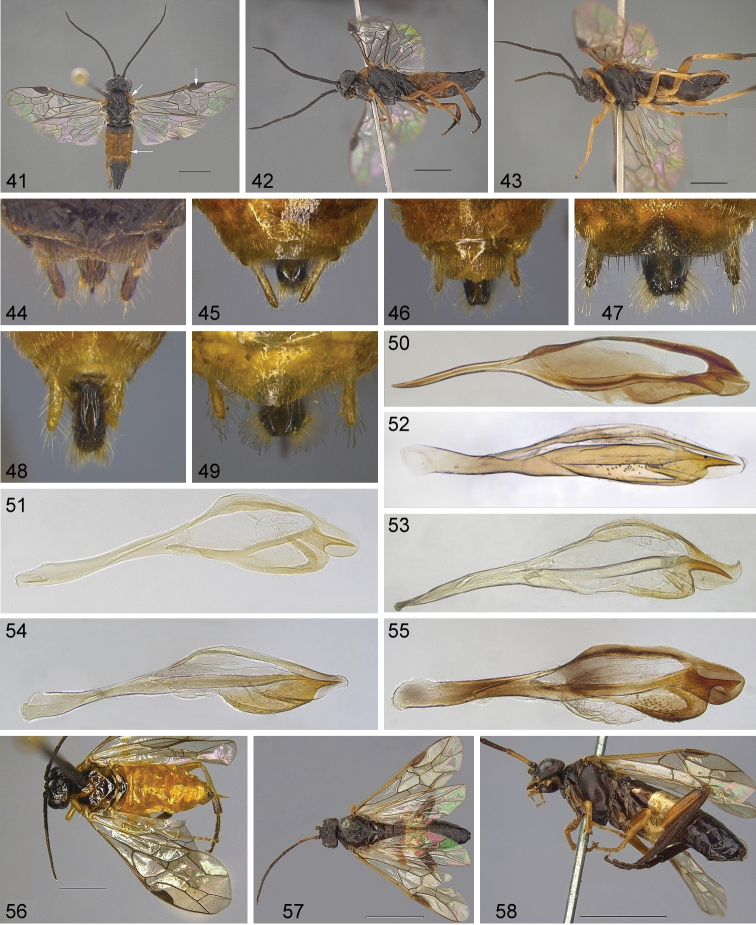
Generic characters of Nematinae**41–42***Nematus lucidus* ♀ **43***N. wahlbergi* ♀ **44***N. wahlbergi* valvula 3 **45***N. umbratus* valvula 3 **46***Euura melanocephalus* valvula 3 **47***E. bohemani* valvula 3 **48***E. ribesii* valvula 3 **49***E. salicis* valvula 3 **50***Nematus wahlbergi* penis valve **51***N. umbratus* penis valve **52***Euura salicis* penis valve **53***E. ribesii* penis valve **54***E. bohemani* penis valve **55***Nematus princeps* penis valve **56***Nematus umbratus* ♀ **57–58***Nematus princeps* ♀. Scale bars: 2 mm (**41–43, 56**), 5 mm (**57–58**)

**Figures 59–68.**
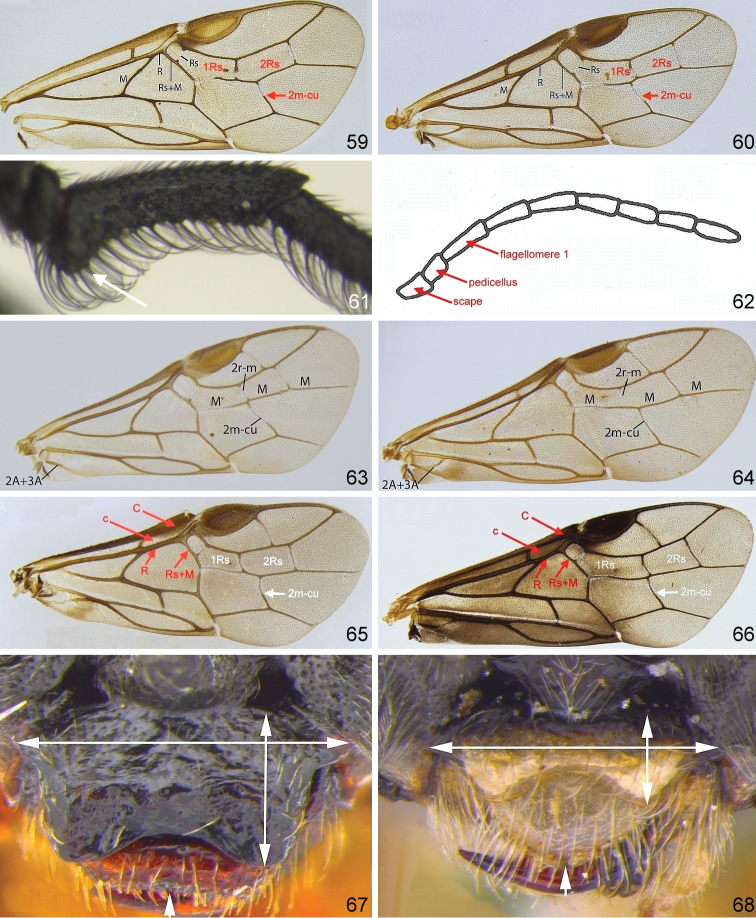
Generic characters of Nematinae**59***Cladius compressicornis* fore wing **60***Platycampus luridiventris* fore wing **61***Cladius ulmi* ♂ flagellomere 1 **62***Armenocampus necopinus* antenna (after Zinovjev 2000) **63***Pseudodineura enslini* fore wing **64***Endophytus anemones* fore wing **65***Hoplocampa chrysorrhoea* fore wing **66***Hemichroa australis* fore wing **67***Neodineura arquata* clypeus **68***Mesoneura opaca* clypeus.

### Key to the West Palaearctic genera and selected species of Nematinae (larvae)

Numbers of setae on dorsal annulets are for only one side of the body, as in Lorenz and Kraus (1957). The best results should be possible with full-grown larvae, but before these undertake a final “extra moult”, in the groups where this applies. Presence or absence of the extra moult is a useful additional taxonomic and identification character in itself (Kontuniemi 1965), but can usually only be scored if the larvae are reared. Larvae of many species which perform an extra moult differ greatly in appearance after this moult from preceding instars: colour pattern and ground-colour frequently change, and setation can be much reduced. Even in species which have no extra moult, pronounced colour differences between instars are often noticeable. Larvae of the monotypic genera *Armenocampus*, *Neodineura*, and *Nescianeura* are unknown, as well as the larvae of many species of *Euura* and *Pristiphora*, particularly the northern species. Even in the less speciose genera, larvae of some species are undescribed, while several others are insufficiently described, or existing descriptions are partly contradictory, e.g., for *Cladius compressicornis* and *brullei*. Because high interspecific morphological variability is already evident in *Euura* larvae, it would not be surprising if larvae were found which have combinations of characters not included in the key. Only the two species of the *Nematus wahlbergi* group known in Sweden are included. Descriptions of larvae of some of the other species of this group may be found in Zinovjev (1979). We have seen no specimens or images of larvae of *Nematus brischkei*: the characters used below to distinguish it are taken from the descriptions by Zaddach (1876) and Chambers (1950). In view of the incomplete and imperfect nature of the available data, the key is highly provisional. Unless otherwise stated, the larvae are exophytic, and feed mostly on leaves. The numbers of species refer to Fennoscandia.

**Table:** 

1	**a** Prolegs present on abdominal segments 2–8 and 10 (Fig. 69), or when (rarely) on 2–7 and 10, then antenna more or less conic, and comprising a single antennomere; **b** Antenna with 1–5 antennomeres, never completely flat; **c** Abdominal segment 3 with 2–6 annulets	**2**
–	**aa** Prolegs present on abdominal segments 2–7 and 10 (Fig. 74); **bb** Antenna with 3–5 antennomeres, sometimes completely flat; **cc** Abdominal segment 3 with 3–6 annulets	**3**
2(1)	**a** Prolegs normally developed on segment 8; **b** Antenna with 1–5 antennomeres; **c** Abdominal segment 3 with 2–6 annulets	[**not Nematinae**]
–	**aa** Prolegs on segment 8 reduced to protuberances much smaller than prolegs on segment 7 (Fig. 69); **bb** Antenna with 3 antennomeres; **cc** Abdominal segment 3 with 6 annulets [*Quercus*]	***Mesoneura opaca***
3(1)	**a** Leaf-miners of Ranunculaceae; **b** Prosternum with median dark fleck and pair of lateral flecks (Fig. 70); dorsum of thorax without any markings (Fig. 71) [Antennae with 3 antennomeres, flat; abdomen segment 3 with 4 dorsal annulets, 2 of which with setae]	***Pseudodineura*** [3 species] **and *Endophytus anemones*** [1 species]
–	**aa** Exophytic on leaves of many plant families, or in galls on *Salix*, fruits of *Ribes* or Rosaceae, or catkins of *Salix*; **bb** Prosternum without dark markings, or only with a median fleck; dorsum of thorax often with markings	**4**
4(3)	**a** Abdominal segment 3 with less than 6 dorsal annulets	**5**
–	**aa** Abdominal segment 3 with 6 dorsal annulets	**24**
5(4)	**a** Abdominal segment 3 with 3–4 dorsal annulets	**6**
–	**aa** Abdominal segment 3 with 5 dorsal annulets	**15**
6(5)	**a** Body flat, woodlouse-shaped (Figs 72–73); **b** Upper anterior head with saddle-shaped indentation (Fig. 73) [*Alnus*]	***Platycampus*** [1 species]
–	**aa** Body at most slightly flattened; **bb** Upper head normal	**7**
7(6)	**a** Supra-anal lobe with pseudocerci (cf. Figs 90–92)	***Euura*** [part: ca. 50 species of *Salix* gall-makers of former *Pontania*, *Phyllocolpa*, *Tubpontania*, and also some exophytic species; overview of galls and larvae of gall-makers in Liston et al. (2017)]
–	**aa** Supra-anal lobe without pseudocerci	**8**
8(7)	**a** Setae on dorsal body annulets arising singly and not from warts (Fig. 74)	**9**
–	**aa** Setae on dorsal body annulets arising from warts, singly or partly in groups (Figs 75–77)	***Cladius* , 10**
9(8)	**a** Dorsal body annulets with some very long setae: as long as length of head (Fig. 74); **b** Abdomen segments with 3 dorsal annulets [*Potentilla fruticosa*, *Dryas octopetala*]	***Pristiphora dasiphorae* and *malaisei*** [former *Pristicampus*]
–	**aa** Dorsal body annulets with short setae: longest much shorter than length of head; **bb** Abdomen segments with 4 dorsal annulets	***Euura*** [part: approx. 16 *Salix* gall-makers of *atra* group; overview of galls and larvae in Liston et al. (2017). Some exophytic species, on various plant genera]
10(8)	**a** Setae on dorsal annulets 2 and 3 of abdominal segment 3 arise in groups from large, pale warts	**11**
–	**aa** Setae on dorsal annulets 2 and 3 of abdominal segment 3 arise singly on small warts which are close to each other (Fig. 75)	***Cladius brullei* , *C. compressicornis***
11(10)	**a** Annulet 1 of abdominal segment 3 with 5–8 setae of which 3–4 arise together from a single wart; **b** Head without black markings (Fig. 76) [Rosaceae: particularly *Rosa*, *Fragaria*, and *Potentilla*]	***Cladius pectinicornis***
–	**aa** Annulet 1 of abdominal segment 3 with 2–5 setae each arising singly from a small wart; **bb** Head at least partly black (Fig. 77) [*Populus*, *Salix*, or *Ulmus*]	**12**
12(11)	**a** Head black (Fig. 77); **b** Surpedal lobe *sometimes* with small black fleck; **c** Anal lobe with large black fleck (Fig. 77) [*Populus* or *Salix*]	**13**
–	**aa** Head green to reddish-yellow with small black flecks; **bb** Surpedal lobe without black markings; **cc** Anal lobe without black fleck [*Ulmus*]	**14**
13(12)	**a** Surpedal lobe with small black fleck; **b** Body of younger instars yellow-green, apart from yellow-orange caudal and distal parts [mature: entirely yellow-orange] [*Populus*, rarely *Salix*]	***Cladius grandis***
–	**aa** Surpedal lobe without small black fleck; **bb** Body of younger instars whitish, apart from yellow-orange caudal and distal parts [*Salix* spp.]	***Cladius aeneus***
14(12)	**a** A black fleck only medially on upper head	***Cladius rufipes***
–	**aa** A black fleck medially on upper head, a pair of black flecks around stemmata, and a black frontal fleck	***Cladius ulmi***
15(5)	**a** Tips of setae on dorsal annulets modified: spatulate or slightly cleft [*Betula*, *Prunus padus*, *Crataegus*, or *Sorbus*: known larvae keyed by Macek (2015)]	***Dineura*** [4 species]
–	**aa** Tips of setae not modified	**16**
16(15)	**a** In female catkins of *Salix* species; **b** Antenna completely flat, comprising several incompletely formed antennomeres (Fig. 78) [Setae on body sparse, very short]	***Euura* [part: ca. 6 species of former *Pontopristia***]
–	**aa** Exophytic on leaves, or endophytic in fruits of Rosaceae; **bb** Antenna completely flat, *or* at least apical antennomere clearly conic	**17**
17(16)	**a** Body somewhat dorso-ventrally flattened (Figs 79–81); **b** Supra-anal lobe with longitudinal keel; **c** Dorsal annulets 1–4 of abdominal segment 3 with setae; d Small head can be withdrawn into prothorax [*Alnus*, *Betula*, or (rarely) *Corylus*]	[*Nematinus*, 6 species], **18**
–	**aa** Body cylindrical (cf. Figs 82–87); **bb** Supra-anal lobe without longitudinal keel; **cc** Dorsal annulets [1–4], or [1, 2 and 4], or [2 and 3] of abdominal segment 3 with setae; **dd** Head normal	**22**
18(17)	**a** Dorsum of body sooty-black; with rows of white warts [*Betula*]	***Nematinus caledonicus***
–	**aa** Dorsum of body green; with or without white warts	**19**
19(18)	**a** Dorsum of body without white warts (Fig. 79) [*Betula*, rarely *Corylus*]	***Nematinus acuminatus***
–	**aa** Dorsum of body with white warts (Figs 80–81)	**20**
20(19)	**a** Top of head with pair of dark brown flecks, one each side of coronal suture (Figs 80–81)	**21**
–	**aa** Top of head without dark brown flecks [*Alnus* spp.]	***Nematinus fuscipennis***
21(20)	**a** Dark brown around orbits, particularly towards temples and rear of head (Fig. 80); **b** Supra-anal lobe dorsally at caudal end with two large dark-brown flecks, often half-moon shaped and partly confluent (Fig. 80) [*Alnus* spp., rarely on *Corylus avellana*]	***Nematinus luteus***
–	**aa** Not dark brown around orbits (Fig. 81); **bb** Supra-anal lobe dorsally without dark-brown flecks (Fig. 81) [*Alnus* spp.]	***Nematinus steini***
22(17)	**a** Dorsum of body with extensive dark pattern of brown patches, or grey longitudinal stripes (Figs 82–83); **b** Dorsal annulets [1, 2 and 4] of abdominal segment 3 with minute setae [On *Larix*]	*** Anoplonyx ***
–	**aa** Dorsum of body at most with small, separate dark markings on abdomen; **bb** Dorsal annulets [2 and 3] or [1–4] of abdominal segment 3 with setae	**23**
23(22)	**a** Dorsal annulets [2 and 3] of abdominal segment 3 with setae; **b** Body without colour pattern except for dark dorsum of abdomen apex (Fig. 84) [In fruits of tree and shrub Rosaceae]	***Hoplocampa*** [9 species]
–	**aa** Dorsal annulets [1–4] of abdominal segment 3 with setae; **bb** Body usually with different colour pattern [Exophytic on leaves, mostly *Salix*]	***Euura*** [part: some former *Amauronematus*]
24(4)	**a** Supra-anal lobe without pseudocerci or protuberances	**25**
–	**aa** Supra-anal lobe with pseudocerci or protuberances	**33**
25(24)	**a** Stipes of maxilla with 0–1 setae	**26**
–	**aa** Stipes of maxilla with 2–3 setae	**29**
26(25)	**a** 3 dorsal annulets [1, 2 and 4] of abdominal segment 3 with setae (Fig. 86)	**27**
–	**aa** 2 dorsal annulets [2 and 4] of abdominal segment 3 with setae	**28**
27(26)	**a** Setae on surpedal and substigmal lobes approx. twice as long as those on body dorsum; **b** All antennomeres incomplete; antenna completely flat [*Populus*, sometimes *Salix*: leaf around larva usually surrounded by pillars of dried white secretion: Fig. 85]	***Stauronematus platycerus***
–	**aa** Setae on surpedal and substigmal lobes not longer than setae on body dorsum (Fig. 86); **bb** Apical 2 antennomeres completely developed; most apical one conic [*Potentilla fruticosa*]	***Pristiphora malaisei*** [see taxon commentary under that name, below]
28(26)	**a** Stipes without setae. If with one seta, then supra-anal lobe in the middle with conspicuous protuberance [coniferous trees, or diverse dicot plants]	***Pristiphora*** [larger part: ca. 90 species]
–	**aa** Stipes with one seta. Supra-anal lobe dorsally with brown-marked depressions [grasses and sedges]	***Euura clitellata* group**
29(25)	**a** Two dorsal annulets [2 and 4] of abdominal segment 3 with setae	***Euura*** [part: *E. spiraeae*, some former *Pachynematus*]
–	**aa** More than 2 dorsal annulets of abdominal segment 3 with setae	**30**
30(29)	**a** Four dorsal annulets [1–4] of abdominal segment 3 with setae	***Euura*** [part: some former *Amauronematus*]
–	**aa** Three dorsal annulets [1, 2 and 4] of abdominal segment 3 with setae	**31**
31(30)	**a** Annulet 1 of abdominal segment 3 with only one seta, annulet 2 without warts bearing several setae	***Euura*** [part: some former *Pachynematus*]
–	**aa** Annulet 1 of abdominal segment 3 with two setae, if not, then annulet 2 with 2 warts each bearing several setae	**32**
32(31)	**a** Body somewhat dorso-ventrally flattened; **b** Annulet 2 of abdominal segment 3 with 4 setae [*Salix*]	***Euura flavescens***
–	**aa** Body cylindrical; **bb** Annulet 2 of abdominal segment 3 with more than 4 setae	***Euura*** [part: some former *Amauronematus*]
33(24)	**a** Caudal margin of supra-anal lobe with 10–12 blunt-conic protuberances; **b** Antenna with 5 antennomeres	**34**
–	**aa** Supra-anal lobe with 2 pseudocerci, and without blunt-conic protuberances; **bb** Antenna with 4 antennomeres	**35**
34(33)	**a** Each body side with three longitudinal black stripes (Fig. 87); **b** Head black [*Alnus*, *Betula*, *Corylus*]	***Hemichroa crocea***
–	**aa** Body without black stripes (Fig. 88); **bb** Head brown (younger larvae), to mainly yellowish-green (older larvae) [*Betula*, *Alnus*]	***Hemichroa australis***
35(33)	**a** Three dorsal annulets [1, 2 and 4] of abdominal segment 3 with setae	**36**
–	**aa** Two dorsal annulets [2 and 4] of abdominal segment 3 with setae	**40**
36(35)	**a** Dorsal annulet 1 of abdominal segment 3 with 1 seta; annulet 2 with 6–7 setae [Surpedal lobe with 8–9 setae; *Picea*]	***Euura insignis***
–	**aa** Dorsal annulet 1 of abdominal segment 3 with 2–6 setae	**37**
37(36)	**a** All antennomeres incomplete and flat [Dorsal annulet 1 of abdominal segment 3 with 2 large and 1 small setae; setae arise from dark flecks]	***Euura*** [part: some former *Amauronematus*]
–	**aa** At least antennomere 4 button-, peg- or cone-shaped	**38**
38(37)	**a** Exophytic on *Lonicera*, rarely on *Symphoricarpos*; **b** Pseudocerci in dorsal view very close to each other, near median line of abdomen (Fig. 90)	**39**
–	**aa** Exophytic on many plant genera, but not *Lonicera* or *Symphoricarpos*; **bb** Pseudocerci in dorsal view much further apart, near lateral edges of tergum (Fig. 92)	***Euura*** [part: former *Pteronidea*]
39(38)	**a** Whole upper head darkened (Fig. 89); **b** A row of dark flecks above the abdominal prolegs (Fig. 89)	***Nematus lonicerae***
–	**aa** Head pale with rather narrow median stripe (Fig. 90); **bb** No row of dark flecks above the abdominal prolegs (Fig. 90)	***Nematus wahlbergi***
40(35)	**a** Substigmal lobe with at least 8 setae	**41**
–	**aa** Substigmal lobe with no more than 6 setae	**42**
41(40)	**a** Pseudocerci apically blunt, and widening towards apex (Fig. 91); distance between them at most 2 × the length of one pseudocercus [*Crataegus*, *Prunus* spp., especially *P. spinosa*]	***Nematus lucidus***
–	**aa** Pseudocerci apically pointed, and cone-shaped; distance between them 3–4 × the length of one pseudocercus [*Salix*, *Rumex*, rarely *Betula*]	***Euura vicina***
42(40)	**a** Abdominal segments ventrally between the prolegs with large black flecks, or body except for more or less pale 1^st^ and last 3 segments nearly completely brown-black (Fig. 93), or abdominal segments with 4 black markings sub- and suprastigmal, and one or more surpedal markings (Figs 95–96)	**43** [*Nematus* part: former *Craesus*]
–	**aa** Abdominal segments without large black flecks ventrally, body markings different [if with black markings, these as more complicated pattern of small flecks: cf. Fig. 92]	**46**
43(42)	**a** Either nearly whole dorsum black (Fig. 93), or each black fleck of uppermost row on body at least as long as half the length of an abdomen segment (Fig. 94); **b** Head nearly entirely black (Figs 93–94)	**44**
–	**aa** Dorsum largely green, more or less with black flecks on sides of body, but individual black flecks much smaller than half the length of an abdomen segment (Figs 95–96); **bb** Head entirely pale: green, to pale brown (Figs 95–96)	**45**
44(43)	**a** At least dorsum of body broadly black, except at most for prothorax and tip of abdomen (Fig. 93) [*Betula*, and *Alnus viridis* in C. Europe]	***Nematus latipes***
–	**aa** Dorsal midline of body entirely without black markings (Fig. 94) [*Betula*, *Alnus*, *Corylus*, *Sorbus aucuparia*, *Carpinus betulus*]	***Nematus septentrionalis***
45(43)	**a** Abdominal prolegs yellow; **b** Coxae entirely pale [*Carpinus betulus*, *Corylus avellana*]	***Nematus brischkei***
–	**aa** Abdominal prolegs green (Fig. 95); **bb** Coxae dark-marked [*Alnus* spp.]	***Nematus alniastri***
46(42)	**a** Pseudocerci visible in dorsal view; subparallel or diverging, and more or less symmetrical [Various plant genera]	***Euura*** [part: former *Pteronidea*]
–	**aa** Pseudocerci not visible in dorsal view; directed inwards, and curved [*Betula*. Body entirely green, except for dark marks on coxae, and small flecks at bases of the more ventral setae: Fig. 97]	***Nematus princeps***

**Figures 69–76.**
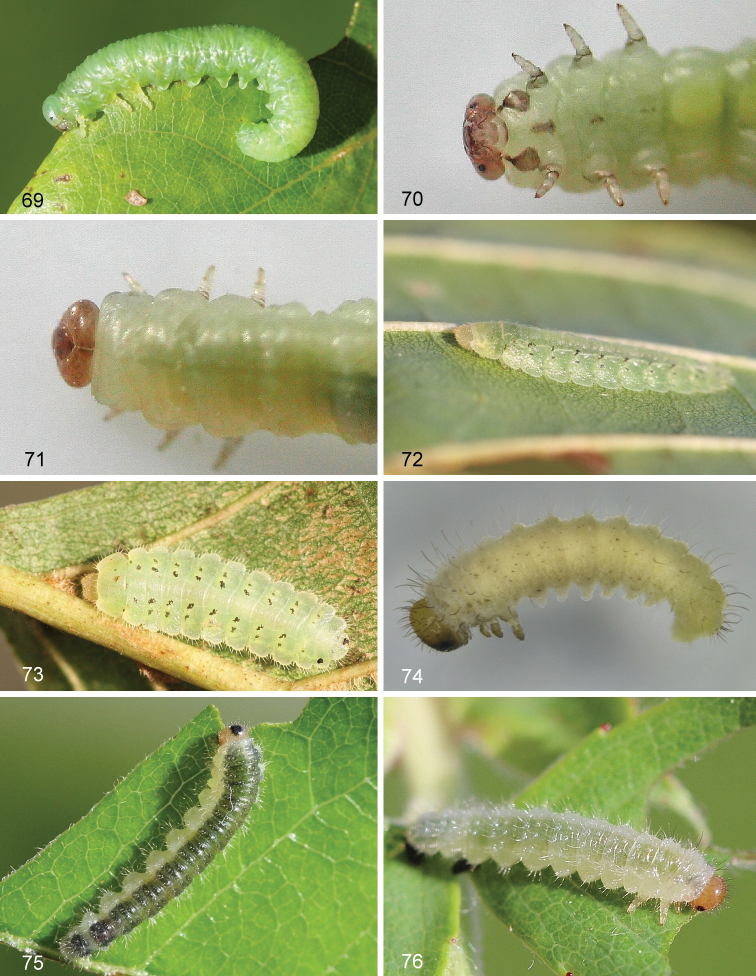
Larvae of Nematinae**69***Mesoneura opaca***70–71***Pseudodineura clematidis*; ventral, dorsal **72–73***Platycampus luridiventris***74***Pristiphora malaisei* from *Dryas octopetala***75***Cladius compressicornis***76***Cladius pectinicornis*.

**Figures 77–87.**
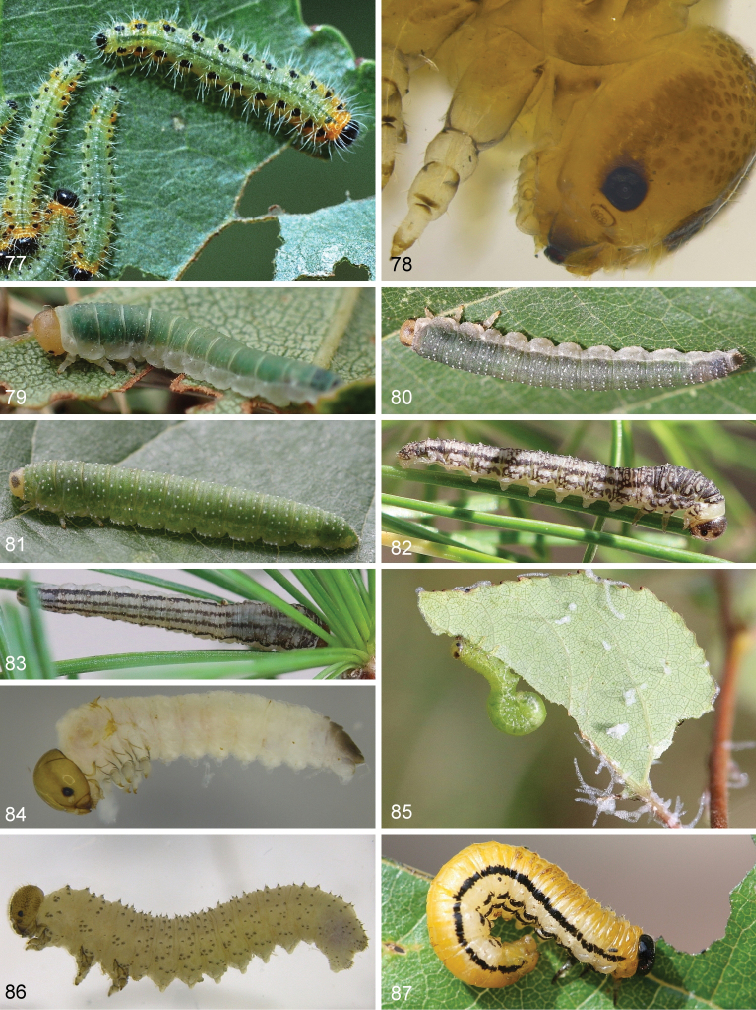
Larvae of Nematinae**77***Cladius grandis***78**Euura sp. amentorum group **79***Nematinus acuminatus***80***Nematinus luteus***81***Nematinus steini***82–83***Anoplonyx albitarsis***84***Hoplocampa crataegi***85***Stauronematus platycerus***86***Pristiphora malaisei* from *Potentilla fruticosa***87***Hemichroa crocea*.

**Figures 88–97.**
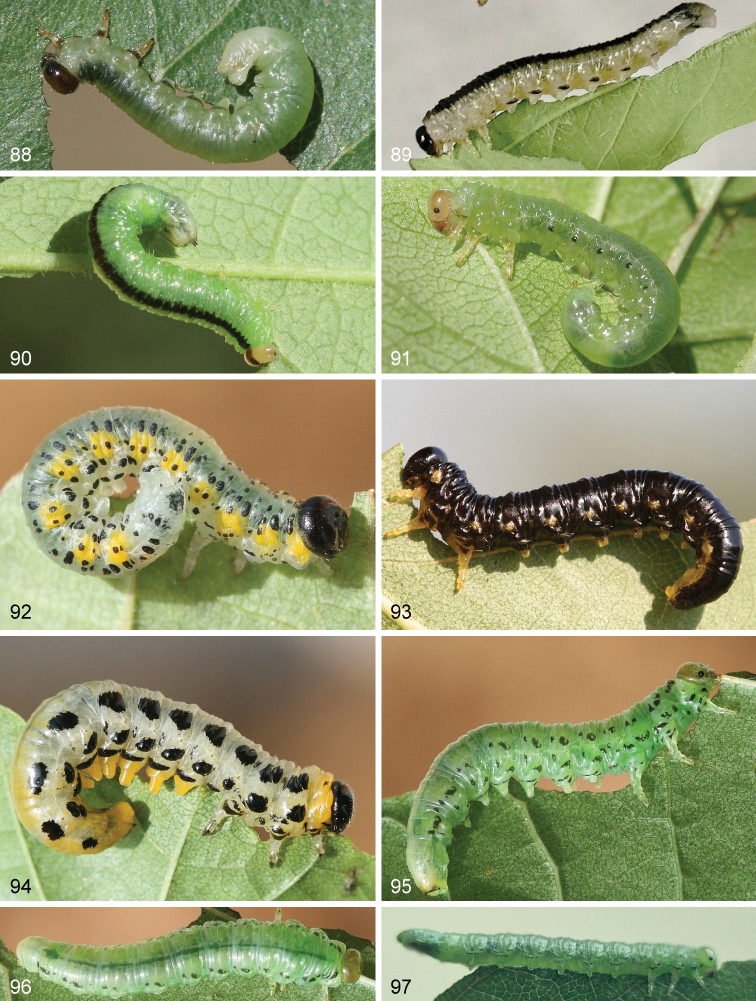
Larvae of Nematinae**88***Hemichroa australis***89***Nematus lonicerae* (photo E. Altenhofer) **90***Nematus wahlbergi***91***Nematus lucidus***92***Euura melanocephalus***93***Nematus latipes***94***Nematus septentrionalis***95–96***Nematus alniastri***97***Nematus princeps* (photo V. Vikberg).

## Taxon commentaries

Synonymy of genus-group names was given by Prous et al. (2014) and is not repeated here, except for *Euura* and *Nematus*, where the synonymy proposed in the former work is extensive, and probably not yet familiar to many users. The known nomina nuda and names for aberrations (unavailable names following International Commission on Zoological Nomenclature (1999)) for the listed species were given by Taeger et al. (2010). Taxa are dealt with in alphabetical order.

### *Anoplonyx* Marlatt, 1896

No reliable key or species treatments are available to date.

### *Armenocampus* Zinovjev, 2000

This genus was erected for a single species, *Armenocampus necopinus* (Zhelochovtsev, 1941), originally described as *Caulocampus necopinus*, known only from the small type series of both sexes collected in Armenia. Nothing is known about its biology.

### *Cladius* Illiger, 1807

No reliable key or species treatments are available to date.

### *Dineura* Dahlbom, 1835

See key and species treatments in Liston et al. (2019a).

### *Endophytus* Hering, 1934

See species treatment in Liston et al. (2019b).

### *Euura* Newman, 1837

Prous et al. (2014) treated a large number of genus-group names as synonyms of *Euura*. A complete list of these is contained therein. The synonyms listed below have been recently used as valid for West Palaearctic taxa. Nearly all species formerly included in these genera, and the majority of species previously placed by many authors in *Nematus*, now belong to *Euura*. The north-west European gall-making species of *Euura* were recently revised by Liston et al. (2017).

*Pontania* Costa, 1852

*Amauronematus* Konow, 1890

*Pachynematus* Konow, 1890

*Pteronidea* Rohwer, 1911

*Pontopristia* Malaise, 1921 (Malaise 1921a)

*Brachycoluma* Strand, 1929

*Decanematus* Malaise, 1931 (Malaise 1931a)

*Pikonema* Ross, 1937

*Phyllocolpa* Benson, 1960 (Benson 1960a)

*Eitelius* Kontuniemi, 1966

*Gemmura* E.L.Smith, 1968

*Eupontania* Zinovjev, 1985

*Larinematus* Zhelochovtsev, 1988

*Polynematus* Zhelochovtsev, 1988

*Bacconematus* Zhelochovtsev, 1988

*Alpinematus* Lacourt, 1996

*Epicenematus* Lacourt, 1998

*Kontuniemiana* Lacourt, 1998

*Lindqvistia* Lacourt, 1998

*Tubpontania* Vikberg, 2010

### *Hemichroa* Stephens, 1835

#### Key to the European species

**Table:** 

1	**a** Female	**2**
–	**aa** Male	**3**
2	**a** Abdomen yellow or orange except for black valvula 3 and more or less tergum 1 (Figs 98, 100); **b** Upper mesepisternum yellow, lower part black (Fig. 100)	*Hemichroa crocea (Geoffroy, 1785)♀
–	**aa** Abdomen black except for more or less red terga 8, 9, 10 and hypopygial area (Figs 99, 101); **bb** Whole mesepisternum black (Fig. 101)	*Hemichroa australis (Serville, 1823)♀
3	**a** Penis valve: upper edge of pseudoceps convex, distal part more evenly tapering; distal projections small (Fig. 107); **b** Parts of abdominal terga and sterna *sometimes* pale (Fig. 102)	*Hemichroa crocea (Geoffroy, 1785) ♂
–	**aa** Penis valve: upper edge of pseudoceps concave, distal part more abruptly tapering; distal projections larger (Figs 104–106); **bb** Abdomen entirely black, except for harpes and more or less distal edge of sternum 9 (Fig. 103)	*Hemichroa australis (Serville, 1823) ♂

**Figures 98–103.**
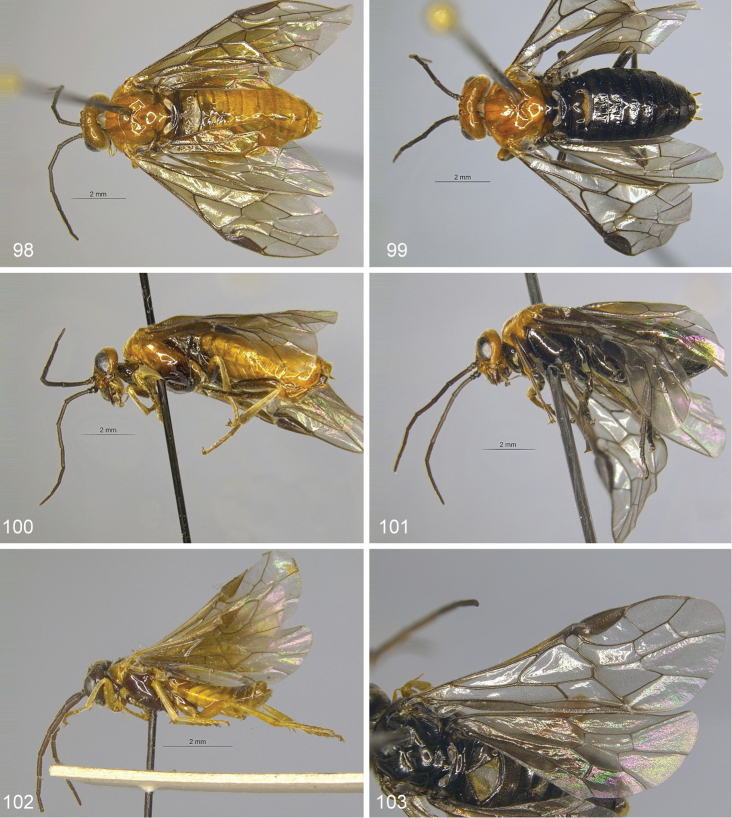
*Hemichroa***98–100***crocea* DEI-GISHym19402 ♀, Germany, Mecklenburg-Vorpommern **99, 101***australis* DEI-GISHym15401 ♀, Sweden, Torne Lappmark **102***crocea* DEI-GISHym31838 ♂, Germany, Mecklenburg-Vorpommern **103***australis* DEI-GISHym20618 ♂, Sweden, Torne Lappmark, fore wing. Scale bar: 2 mm.

#### Hemichroa australis

Taxon classificationAnimaliaHymenopteraTenthredinidae

(Serville, 1823)

701E422AD8EE5E85913C78C9556E76E5

Tenthredo alni Linné, 1767: 925. Lectotype ♀, designated by Malaise and Benson (1934: 8), not examined, in LSUK (images: http://linnean-online.org/16581/). Type locality: Sweden. Primary homonym of Tenthredo alni Linné, 1758 (Nematus septentrionalis (Linné, 1758)).
Tenthredo luctuosa Hill, 1773: 5–6, pl. 1. Syntype(s) ♀, lost. Type locality: Uxbridge (United Kingdom). Treated as nomen oblitum and synonymised with australis by Blank et al. (2009: 32).
Tenthredo australis Serville, 1823: 16. Syntype(s) ♀, lost. Type locality: Midi (France). Nomen protectum, as stated by Blank et al. (2009: 32).
Tenthredo australis Lepeletier, 1823:71. Syntype(s) ♀, lost. Type locality: Midi (France). Primary homonym of Tenthredo australis Serville, 1823.
Hemichroa monticola Ermolenko, 1960: 208–210. Holotype ♀ (Schmalhausen Institute, Kiev: not examined) and 4 female paratypes (one examined). Type locality: Ukraine, Lvovskoj oblasti, Slavekogo rajona, Tuhovalskom perevale. **Syn. nov.**

##### Taxonomy.

Ermolenko (1960) stated that *australis* differs from *monticola* in the following characters [character state for *monticola* in brackets]:

– lower surface of antenna noticeably paler than the upper [uniformly dark]

– medial emargination of clypeus deep, usually exceeding half of its length [reaching half of its length]

– intercostal and lanceolate cells of the fore wing and main half of the hind wing are clearly darkened [wings nearly completely hyaline]

– the 2^nd^ anal cell of the posterior wing is almost equal to the length of the median cells [2^nd^ anal cell of the posterior wing noticeably shorter than median one]

– 9^th^ tergum predominantly dark [9^th^ tergum red]

– cerci yellow [cerci basally yellow, apically fuscous]

– valvula 3 of ovipositor on lower margin noticeably convex in lateral view [only slightly convex]

– teeth of the proximal half of the ovipositor have two or more smaller additional denticles at the base [these teeth with only one small additional tooth]

Only a single paratype of *monticola* was available for examination, but we also examined four females (HNHM) which have the combination of colour characters described for *monticola* and were collected at subalpine levels in the Ukrainian Carpathians, as was the type series of *monticola*. We did not observe any significant difference in the depth of the clypeal emargination between Carpathian specimens and *australis* from other parts of Europe. The other characters used to distinguish *monticola* are either extremely weak, such as the slightly darkened tips of the cerci and the degree of curvature of the lower edge of valvula 3, or are variable among studied *australis* females, such as the length of the hind wing anal cell and the presence or absence of denticles on the more basal serrulae of the lancet (Figs 108–111). The shape of sawteeth and the number of serrulae can even vary between the left and right lancets of the same individual (Figs 108–109), possibly as a result of wear (see Schmidt and Walter 1995). Ermolenko considered *H. monticola* to be a neo-endemic element of the Carpathian subalpine fauna, associated with *Alnus viridis*, but several of the characters which he gave as distinguishing it from *australis* occur apparently independently of each other in the *australis* females which we have examined from many parts of the West Palaearctic. For example, tergum 9 mainly pale, but whole wing-membrane blackish from base of fore wing up to approximately the level of the pterostigma [Germany, Berlin], or antennae entirely black, and wing membrane nearly entirely hyaline, but 9^th^ tergum black [Sweden, Lapland]. In our opinion, Ermolenko underestimated the range of variability in *australis*, and *monticola* falls within this range. Therefore, we treat the taxa as conspecific. Nevertheless, comparison of relevant genetic data should still be undertaken.

**Figures 104–107.**
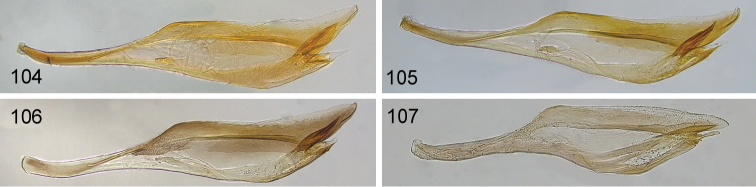
*Hemichroa*, penis valves **104***australis* DEI-GISHym15392 Germany, Saxony **105***australis* DEI-GISHym20618, Sweden, Kiruna **106***australis* DEI-GISHym84982, Japan, Honshu **107***crocea* DEI-GISHym31838, Germany, Mecklenburg-Vorpommern.

**Figures 108–112.**
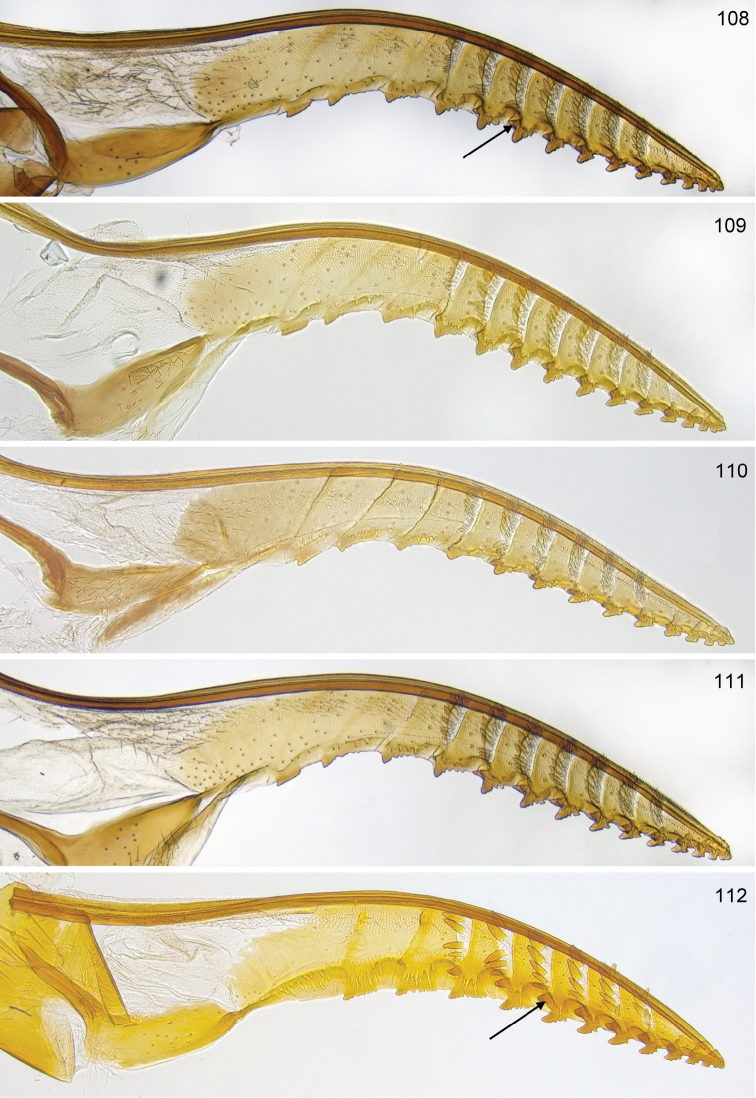
*Hemichroa*, lancets **108–109***australis* DEI-GISHym15387, Sweden, Torne Lappmark; arrow, spurette **110***australis* DEI-GISHym31836, Ukraine, Carpathians **111***australis* DEI-GISHym31837, Russian Federation, Baskiria **112***crocea* DEI-GISHym19401, Germany, Brandenburg; arrow, spurette.

Previously published descriptions of the male of *Hemichroa australis*, and the colour characters which are claimed to distinguish it from that of *crocea*, are partly contradictory, and may not be reliable. Enslin (1915: 317) wrote [translated from German]: “According to Cameron, the male of *H. crocea* Geoffr. is just like that of *H. alni* [*australis*]; Cameron (Monograph Brit. Phyt. Hym. II p. 7) saw some males of *crocea* reared by Fletcher and could not distinguish them from *H. alni*. Because nothing further on this subject is reported in the literature and it was not possible for us to obtain males of *H. crocea* for examination, the separation of the males of these species must remain unresolved until a later date”. Benson (1958) stated that the male of *australis* “Differs from *crocea* ♂ in that the antenna is at least red below [*crocea*: antenna entirely black] and the stigma of the wing is piceous [*crocea*: pterostigma brown in the middle] “. Smith (1975), in his key to World *Hemichroa* species, wrote that he did not know the male of *australis*, and repeated the characters given by Benson (1958). But in the text under *H. crocea*, Smith (1975) wrote “It may be separated from other species by the presence of the radial crossvein [2r-rs] in the fore wing and characters of the genitalia (figs 3, 4)”. The first character state was surely mentioned in error: all *Hemichroa* species usually possess vein 2r-rs, except for the taxon treated by Smith (1975) as *H. militaris* (Cresson, 1880), which is currently placed in *Dineura* (Fig. 1, Prous et al. 2014). See below under *crocea* for additional discussion of diagnostic characters of males of *australis* and *crocea*.

##### Description.

Body length: female 6.5–8.5 mm, male 6.0–6.5 mm. Wing colour highly variable in both sexes, from nearly entirely hyaline, to entire hind wing and basal fore wing up to about pterostigma conspicuously darkened. Female (Figs 99, 101): Black. Red are head, except more or less for labrum and antenna; pronotum, tegula, mesoscutum, more or less mesoscutellar appendage; more or less the apex of abdomen. Legs black, except for more or less brownish fore legs. Lancet: Figs 106–109. Male (Fig. 103): Head and body entirely black, except more or less for underside of antennae, tegulae, extreme upper posterior edge of pronotum, and subgenital plate. Legs entirely red, except for black coxa and more or less trochanters and trochantelli. One male (DEI-GISHym20617), presumably atypical, has the thorax red and black patterned, exactly as in females. Penis valve: Figs 104–106; note the variability in shape of the distal projections.

Our characterisation of the male of *australis* is based primarily on three specimens from Germany (BC ZSM HYM 04094), Lapland (DEI-GISHym20618), and Japan (DEI-GISHym84982), with identity confirmed by barcoding. Fore wing basally darkened or mostly subhyaline, the antennae black with reddish undersides (or nearly completely pale in the Japanese specimen), and the stigma uniformly dark. The body is completely black, except for the slightly brown tegulae, harpes, and distal edge of sternum 9; and all tibiae completely pale. One further male from Torne Lappmark in the SDEI, and the long series of males from Ukraine, have the same coloration except for mostly subhyaline fore wing. The latter exhibit little variability, except that the tegulae and upper posterior edges of the pronotum may be completely black, or more or less brown, and the antennae usually extensively reddish, but occasionally nearly completely black. The wing veins of the males from Lapland, including the fore wing pterostigma, are, however, darker than the Ukrainian specimens.

##### Similar species.

See key, and notes on male (above, and under *crocea*, below). Compared with *crocea* (Fig. 112), the most obvious differences in the lancet of *australis* (Figs 108–111) are the greater number and smaller size of ctenidia on the annular sutures, smaller distance between each basal and median sawtooth and its spurette, and its less hooked median sawteeth.

##### Life history.

Host plants (in Europe): *Betula pendula*, *pubescens* (Kontuniemi 1960), *pubescens* var. pumila (see Specimens examined), *utilis* (Schedl 2010), *Alnus glutinosa*, *incana*, and *viridis* (Kontuniemi 1960, Pschorn-Walcher and Altenhofer 2000), and further *Alnus* species in the East Palaearctic. Larvae solitary, and cryptic (Fig. 88). Boevé (2015) compared the defensive strategy of *australis* and *crocea* larvae. Two overlapping generations in the lowlands. Although males of both European *Hemichroa* species have generally been considered to be rare (e.g., Benson 1958, Smith 1975), males of *australis* are, at least regionally, evidently rather abundant. In a series of 104 specimens collected by Ermolenko in the montane zone of the Ukrainian Carpathians, 92 are males, and 2 of 5 specimens recently collected in the Torne Träsk Region are males. Malaise (1921b) also noted that although males of *australis* are usually extremely rare, three of six specimens which he collected in the Torne Träsk area were males. Perhaps males are more frequent in areas with a cooler climate, which would represent an interesting departure from the usual pattern in Tenthredinoidea of a higher female to male ratio in warmer areas (Benson 1950: 126).

##### Distribution.

Trans-palaearctic from the British Isles, through north and central Europe (Taeger et al. 2006) to Yakutia (Sundukov 2017) and Japan (Smith 1975; see also Specimens examined).

##### Occurrence in Sweden.

Published records: Skåne (Andersson 1962), “this species seems to be widespread throughout Sweden” (Thomson 1871). Material was examined from Skåne, Småland, Östergötland, Bohuslän, Uppland, Västmanland, Jämtland, Lycksele Lappmark, Torne Lappmark.

##### Specimens examined.

Czech Republic: 1♀ (ZSM). France: Gironde: 1♂ (DEI-GISHym20617), Saucats, 44.65000N, 0.60000W, 16.08.2012, leg. H. Chevin (SDEI). Germany: 17♀ (SDEI, ZSM, ZMHB). 1♂ (DEI-GISHym31923), Bayern, Dingolfing, Stadtwald, 06.06.1992, leg. Liston (SDEI). 1♂ (DEI-GISHym15392), Sachsen, Erzgebirge, Altenberg Umg., 22.07.1985, leg. S. Walter (SDEI). Japan: Honshu: 1♂ (DEI-GISHym84982), Omeshidake W, Road 112, 1900 m, 36.62400N, 138.45400W, 22.07.2016, leg. A. Taeger (SDEI). Russia: Respublika Bashkortostan (Baskiria): 1♀ (DEI-GISHym31837), Burzyanskaya obl. / Baskir Reserve, 53.16666N, 57.50000E, 30.06.1985, leg. V. M. Ermolenko (HNHM). Primorskiy Kray: 1♀, Anisimovka: Gribanovka 1km N, 450 m, 43.12600N, 132.79700E, 18.06.2017, leg. A. Taeger (SDEI). Sweden: Skåne : 1♀ (NHRS-HEVA000006494), no exact locality, leg. Boheman (NHRS). 1♀, Krankesjön, 55.70000N, 13.46666E, 03.08.1974, leg. H. Andersson (MZLU). Småland: 2♀ (NHRS-HEVA000006495–6), no further data (NHRS). 1♀ (NHRS-HEVA000006500), no further data (NHRS). Östergötland: 1♀ (NHRS-HEVA000006498), no exact locality, leg. Wahlgren (NHRS). Bohuslän: 1♀ (NHRS-HEVA000006499), no further data, leg. Boheman (NHRS). Uppland: 1♀ (NHRS-HEVA000003425), Frescati, leg. Malaise (NHRS). 1♀ (NHRS-HEVA000006502), Ulleråkers sjukhus (Asylen) (NHRS). Västmanland: 1♀, Sala kommun, Nötmyran (Västerfärnebo), birches at Islingby, Östermyran, 59.94198N, 16.30944E, 25.10.2003–08.06.2004, leg. SMTP (NHRS). Jämtland: 1♀ (NHRS-HEVA000006501), no further data (NHRS). Lycksele Lappmark: 2♀ (NHRS-HEVA000006503–4), Sorsele, 29.07.1929 and 05.07.1931, leg. Gaunitz (NHRS). Torne Lappmark: 3♀ (NHRS-HEVA000006505, 6507, 6508), Torne Träsk, 04/06.07.1918 and one without date, leg. Malaise (NHRS). 2♂ (NHRS-HEVA000006510/12), Abisko, 04/08.07.1918, leg. Malaise (NHRS). 1♂ (NHRS-HEVA000006511), Torneträsk, 03.07.1918, leg. Malaise (NHRS). 1♂ (NHRS-HEVA000006513), Kummavuopio, 23.07.1923, leg. Bruce (NHRS). 1♂ (DEI-GISHym20618), Kiruna nr. airport, 450 m, 67.84000N, 20.35000E, 21.06.2012, leg. Liston & Taeger (SDEI). 2♀ (DEI-GISHym15387, 15401), Kiruna nr. airport, 450 m, 67.84000N, 20.35000E, 01.07.2012, leg. Liston & Taeger (SDEI). 1♂, Abisko National Park, E10, 390 m, 68.35300N, 18.81500E, 30.06.2012, leg. Liston & Taeger (SDEI). 1♀, Abisko 9 km E (Stordalen), 400 m, 68.35000N, 19.03500E, 04.07.2016, leg. Liston & Prous (SDEI).1♀, Abisko 6 km W, 650–900 m, 68.34200N, 18.69100E, 02.07.2016, leg. Liston & Prous (SDEI). 1♀, Kiruna, near airport, 450 m, 67.84000N, 20.35000E, 22.06.2016, leg. Liston (SDEI). 1 larva (DEI-GISHym83694), on Betula pubescens var. pumila, Abisko 9 km E (Stordalen) (Sweden: Norrbottens Län), 400 m, 68.35000N, 19.03500E, 05.08.2017, leg. Liston & Prous (SDEI). Switzerland: 3♀ (SDEI, ZSM). Ukraine: 12♀, 92♂ (HNHM), and: 1♀ (DEI-GISHym30203: Paratype of *H. monticola* Ermolenko), Lvivska Oblast, Slavekogo rajona, Tukhovalsky Pass, 16.08.1957, leg. V. M. Ermolenko (ZISP). 1♀ (DEI-GISHym31836), Ivano-Frankivs’ka Oblast’, Csernogora, Pozsizsevszkaja, 26.06.1975, leg. V. M. Ermolenko (HNHM).

#### Hemichroa crocea

Taxon classificationAnimaliaHymenopteraTenthredinidae

(Geoffroy, 1785)

8032C281BB435E298F91E1C4133530E6

Tenthredo crocea Geoffroy in Fourcroy, 1785: 364. Syntype(s) ♀, lost. Type locality: Paris (France).
Tenthredo rufa Panzer, 1799: 72:2. Syntype(s) ♀, lost. Type locality: Germany. Primary homonym of Tenthredo rufa Retzius, 1783.
Hemichroa stigma Stephens, 1835: 56. Syntype(s) ♀, most likely lost. Type locality: Ripley (United Kingdom). Listed in synonymy with Hemichroa rufa (Panzer) by Dalla Torre (1894: 283).
Leptocercus nigriceps Thomson, 1871: 78. Holotype ♀, not examined, in MZLU. Type locality: Skåne (Sweden). Synonymy with crocea by Lindqvist (1954).
Dineura (Leptocera) unicolor Rudow, 1872: 218. Syntype(s) ♀, most likely lost. Type locality: not given [Germany]. Synonymy by Konow (1897: 259).
Dineura americana Provancher, 1882: 292–293. Holotype ♀, not examined, ULQC. Type locality: Chicoutimi (Canada). Synonymy by Ross (1937: 79).
Nematus ardens Zaddach in Brischke, 1883a: 133–134. Holotype ♀, lost. Type locality: Carolath (Siedlisko, Poland). Listed in synonymy by Konow (1905: 49).
Dineura pallida Ashmead, 1890: 15. Holotype ♀, not examined, in USNM. Type locality: West Cliff, Ca. (USA). Synonymy by Ross (1937: 79).
Hemichroa dyari Rohwer, 1918: 170–171. Holotype ♀, not examined, in USNM. Type locality: Woods Hole, Massachusetts (USA). Synonymy by Ross (1937: 79).
Hemichroa (Hemichroa) orientalis Rohwer, 1921: 108–109. Holotype ♀, not examined, in USNM. Type locality: Kumaon, Ramgark (India). Synonymy by Smith (1975: 298).
Hemichroa (Hemichroa) washingtonia Rohwer & Middleton, 1932: 97–98. Holotype ♀, not examined, in USNM. Type locality: Seattle, Washington (USA). Listed in synonymy by Ross (1937: 79).

##### Description.

Body length: female 5.5–8.5 mm, male 5.5 mm (only one examined). Female (Figs 98, 100): Orange-red. Black are (more or less): labrum, propleuron, mesopleuron, metapleuron, metanotum, ventral part of mesepistermum, abdominal tergum 1, valvula 3. Coxae, trochanters and femora brown, with variable black markings. Tibiae basally pale (whitish), apically dark. Tarsi dark. Lancet: Fig. 112. Male (Fig. 102): Head including antennae, and body black, except more or less for tegulae, pronotum, and parts of abdominal terga and sterna. Legs red, except for darkened coxa, more or less trochanters and trochantelli, metatarsus, and apex of metatibia. Penis valve: Fig. 107.

We have only examined one old male specimen (DEI-GISHym31838), without genetic data, which we think belongs to *crocea*, because of the similarity of its penis valve to that illustrated by Smith (1975; fig. 4) as *crocea*, and differences in the penis valves of *australis* identified by us, using sequence data. This *crocea* male has its abdomen and parts of the mesoscutum extensively yellow, but completely black antennae, as well as darkened metatarsus and metatibia apex. However, the original descriptions of the males of *Hemichroa dyari*, *pallida* and *washingtonia* (Rohwer 1918, Rohwer and Middleton 1932), all of which are currently treated as synonyms of *H. crocea*, indicate that body colouration is variable, and can be as dark as in male *australis*. The metatibia and metatarsus may apparently also be dark or pale, as respectively described by Rohwer (1918) for males of *dyari* and *pallida*. On the other hand, the descriptions of North American *crocea* males suggest that the antennae are completely dark, as described by Benson (1958) for European males.

##### Similar species.

See key and notes on *australis*, above.

##### Life history.

Host plants: *Alnus glutinosa*, *incana*, *viridis*, *Betula pendula*, and sometimes *Corylus avellana* (Pschorn-Walcher and Altenhofer 2000). *Salix* is mentioned repeatedly in various works as a host, but no unambiguous original record of feeding by larvae on *Salix* has been located. Larvae gregarious, and brightly coloured (Fig. 87). Boevé (2015) compared the defensive strategy of *crocea* and *australis* larvae. Usually two overlapping generations in the lowlands (Hopping 1937, Pschorn-Walcher and Altenhofer 2000), but mainly univoltine at subalpine levels (Kriegl 1964). Whereas the subalpine populations are entirely parthenogenetic (Kriegl 1964), approximately 3% males were reared in northern Germany (Pschorn-Walcher and Altenhofer 2000).

##### Distribution.

Found widely in the Holarctic, from the British Isles, through central and northern Europe (Taeger et al. 2006), to the Russian Far East (Sundukov 2017), Japan, northern India (Smith 1975), reaching into the Oriental Region in China (see Specimens examined), and transcontinental in North America (Smith 1975). According to Ross (1932), *Hemichroa crocea* was probably introduced to North America, but Kriegl (1964) concluded that the species occurs there naturally, because a similar assemblage of parasitoid species is found in Europe and North America.

##### Occurrence in Sweden.

Published records: Skåne (Andersson 1962), “sparingly, but distributed from Skåne to Lapland” (Thomson 1871). Material was examined from Skåne, Småland, Öland, Gotska Sandön, Södermanland, Dalarna, Lappmark.

##### Specimens examined.

Canada: Quebec: 1♀ (DEI-GISHym15340), Gatineau Park 1.8km N Eardley, Juniperus virginiana stand, 60–80 m, 45.56667N, 76.09139W, 31.08.–07.09.2012, leg. CNC Hymenoptera Team (SDEI). China: Sichuan: 1♀ (DEI-GISHym17831), Gongga Shan, 2200 m, 29.59700N, 102.05000E, 29.06.2009, leg. Blank, Liston & Taeger (SDEI). Germany: Baden-Württemberg: 1♀ (SDEI). Bayern: 4♀ (BC ZSM HYM 04090, 04091, 16633, 16740) (ZSM). Berlin: 1♀ (SDEI). Brandenburg: 1♀ (DEI-GISHym19401) (SDEI). Hessen: 1♀ (DEI-GISHym17970) (SDEI). Mecklenburg-Vorpommern: 1♀ (DEI-GISHym19402) (SDEI). 1♂ (DEI-GISHym31838), Kalkhorst near Neustrelitz, 53.31666N, 13.06666E, 27.06.1884, leg. F. W. Konow (SDEI). Nordrhein-Westfalen: 1♀ (SDEI). Sachsen: 1♀ (SDEI). Portugal: Viana do Castelo: 1♀ (DEI-GISHym19668), Monção 10 km E, 30 m, 42.08658N, 8.36285W, 09.05.2012, leg. Blank, Jacobs, Liston & Taeger (SDEI). Sweden: Skåne : 1♀ (NHRS-HEVA000006485), leg. Boheman (NHRS). Småland: 1♀ (NHRS-HEVA000006489), Kalmar, 05.1919, leg. Hedgren (NHRS). Öland : 1♀ (NHRS-HEVA000003424), Stora Rör, 08.08.1941, leg. Wieslander (NHRS). Gotska Sandön: 1♀ (NHRS-HEVA000006487), leg. Jansson (NHRS). Södermanland: 1♀ (NHRS-HEVA000006488), Drevviken, leg. Smidt (NHRS). Dalarna: 1♀ (NHRS-HEVA000006486), “Dalecarlia alpina”, leg. Boheman (NHRS). Middle and southern Lapland: 1♀ (NHRS-HEVA000006491), “Lapponia meridionalis”, leg. Boheman (NHRS). 1♀ (NHRS-HEVA000006492), “Lapponia intermedia”, leg. unknown (NHRS).

### *Hoplocampa* Hartig, 1837

See key and species treatments in Liston et al. (2019c).

### *Mesoneura* Hartig, 1837

Only two species are known from the West Palaearctic (Liston 2012), and only *M. opaca* occurs in north-west Europe. The nominal taxon described as Tenthredo (Selandria) umbrosa Eversmann, 1847 was treated in several works (e.g., Dalla Torre 1894, Konow 1905, Taeger et al. 2010) as a third, valid West Palaearctic *Mesoneura* species, but examination of the type revealed it to be a male specimen close to *Euura clitellata* (Serville, 1823).

### Key to West Palaearctic species, based on Liston (2012):

**Table:** 

1	**a** Females	**2**
–	**aa** Males	**3**
2(1)	**a** Upper side of abdomen mainly black; at least with a continuous black dorsal vitta (Fig. 113); **b** Lancet with 14–15 annuli; serrulae, particularly basal ones, rather flat (Fig. 115)	*Mesoneura opaca ♀
–	**aa** Upper side of abdomen mainly yellow apart from black 1^st^ tergum and some black lateral spots (Fig. 114); **bb** Lancet with ca. 20 annuli; serrulae prominent, hooked (Fig. 116)	*Mesoneura lanigera ♀
3(1)	**a** Abdominal terga 5–8 with a deep, sharply delimited medial depression edged with a row of long setae (Fig. 117); **b** All terga mainly black, except for more or less pale extreme apical margins; **c** Apical margin of sternum 9 medially slightly produced (Fig. 117); **d** Length 6.5–8.0 mm	***Mesoneura opaca*** ♂
–	**aa** Abdominal terga 5–8 with at most a shallow, ill-defined medial depression, without row of modified setae along edge (Fig. 118); **bb** Terga 2–4 entirely yellow-brown (Fig. 118); **cc** Apical margin of sternum 9 truncate or medially even slightly emarginate (Fig. 118); **dd** Length 5.5–6.5 mm	***Mesoneura lanigera*** ♂

**Figures 113–118.**
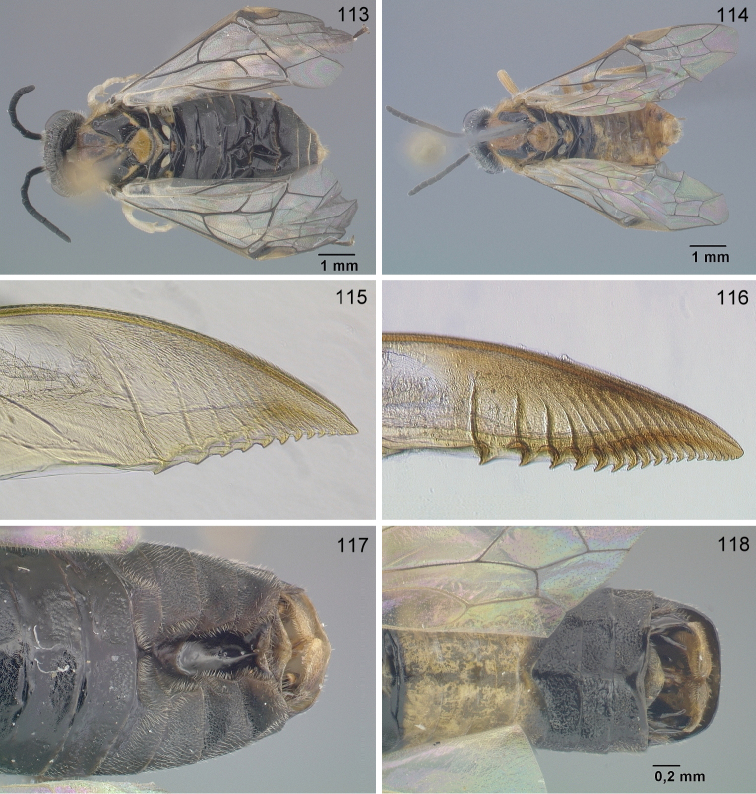
*Mesoneura***113***opaca* ♀ DEI-GISHym17936 **114***lanigera* ♀ DEI-GISHym17933 **115***opaca* DEI-GISHym17935 lamnium of lancet **116***lanigera* DEI-GISHym17933 lamnium of lancet **117***opaca* ♂ DEI-GISHym17937 abdomen apex **118***lanigera* ♂ DEI-GISHym17934 abdomen apex.

#### Mesoneura opaca

Taxon classificationAnimaliaHymenopteraTenthredinidae

(Fabricius, 1775)

B7E2DA0E8B6E5739A425C75D840801BB

Tenthredo opaca Fabricius, 1775: 323. Syntype(s) ♀, Suecia, lectotype ♀ here designated (ZMUC-GISHym1061), in ZMUC. Type locality: Sweden. Remarks. Lectotype labeled “opaca”, “ZMUC-GISHym1061”. Right antennal flagellomeres 6–7 and fore tarsomere 5 missing. In the lectotype the distal section of the posterior anal vein (2A) is absent on the hind wing and thus the anal cell (A) widely open distally. Otherwise it corresponds with the current concept of Mesoneura opaca, which is quite variable in coloration. This specimen has the median mesoscutal lobes red on both the medial and the lateral edges, and the mesoscutellum black.
Tenthredo (Allantus) verna Klug, 1816: 55–56. Syntypes ♀, Berlin, in ZMHB. Type locality: Berlin (Germany). Synonymy with Tenthredo opaca Fabricius, 1775 by Klug (1819: 81). Remarks.In ZMHB are 7 ♀ with the collection catalog number 13747 (GBIF-GISHym2504 to 2510). This number means: [identification:] Tenthredo opaca Fabr.; [specimens:] 8.; [locality, collector:] German. Kl.; Dania - Drewsen. Therefore, these specimens were collected in Germany or Denmark, and their unequivocal identification as syntypes (from Germany) is impossible. Images of GBIF-GISHym2504: https://doi.org/10.6084/m9.figshare.4774588).
Tenthredo punctigera Serville, 1823: 103. Lectotype ♀, designated by Lacourt (2000: 103) not examined, in MNHN. Type locality: Paris (France). Synonymy (for Tenthredo punctigera Lepeletier, 1823) with Dineura opaca (Fabricius, 1775) by Hartig (1837: 229).
Tenthredo punctigera Lepeletier, 1823: 110. Lectotype ♀, designated by Lacourt (2000: 103) not examined, in MNHN. Type locality: Paris (France). Synonymy with Dineura opaca (Fabricius, 1775) by Hartig (1837: 229). Primary homonym of Tenthredo punctigera Serville, 1823.
Selandria biloba Stephens, 1835: 54. Syntype(s) ♀, not examined, in BMNH. Type locality: London (United Kingdom). Synonymy by Kirby (1882: 157).
Dineura (Mesoneura) pallipes Hartig, 1837: 229. Syntype(s) ♀, most likely lost. Type locality: Harz (Germany). Synonymy by Cameron (1875: 252). Remarks. There are three females under Dineura pallipes Hartig in the collection of Saxesen, one labelled “Hartig!”. However, these specimens do not fit Hartig’s description.
Dineura dorsalis Förster, 1844: 263. Holotype ♀, most likely lost. Type locality: Aachen (Germany). Synonymy by Cameron (1875: 252).
Mesoneura opaca var. nigerrima Enslin, 1914: 271. Syntype(s) ♀, no data, lectotype ♀ here designated (GBIF-GISHym3158, images: https://doi.org/10.6084/m9.figshare.4775329), in ZSM. Type locality: Südtirol (Italy).
Mesoneura opaca var. lucida Enslin, 1914: 271. Syntype(s) ♀, no data, most likely lost. Type locality: Europe.
Mesoneura opaca var. obscuriventris Enslin, 1914: 271. Syntype(s) ♀, no data, lectotype ♀ here designated (GBIF-GISHym3160, images: https://doi.org/10.6084/m9.figshare.4775341), in ZSM. Type locality: Erlangen (Germany).

##### Description.

Body length: female 5.5–9.0 mm, male 6.5–8.0 mm. Female (Fig. 113): head including antenna black, except for white clypeus and labrum, and sometimes brown flecks on interantennal area / just dorsal of toruli / lower outer orbits. Thorax black. In darkest specimens only pronotum and tegula pale. Palest specimens with yellow-brown whole median mesoscutal lobe, parts of lateral lobes, mesoscutellum and appendage, upper mesepisternum, and parts of metanotum. Fore wing pterostigma completely pale, to pale in middle with darkened edges. Legs pale, with coxae, femora and apical tarsomeres more or less darkened. Abdomen from completely black, to completely pale on underside with lateral parts of terga more or less pale, and pale tergum 10 and cerci. Lancet: Fig. 115. Male (only four examined): Black; only ventral parts of clypeus pale, labrum pale to nearly completely dark. Thorax at most with pale edges of pronotum, and more or less tegulae. Leg colour similar to female, but darkest males with apex of metatibia darkened, and palest with tarsi completely pale. Abdomen black except for brownish narrow distal margin of sternum 9 and more or less harpes, and sometimes around the depressed parts of terga 5–8. Penis valve: Liston (2012: fig. 4) [not distinguishable from that of *lanigera*].

##### Similar species.

In the West Palaearctic, only *Mesoneura lanigera* Benson, 1954 (south-east Europe, Transcaucasus and Cyprus) could be mistaken for *opaca*: see key.

##### Life history.

Host plants: *Quercus* species, including *robur* (Pschorn-Walcher and Altenhofer 2000), *pubescens*, and *rubra* (Liston 2011). Univoltine species. Oviposition in the leaf midrib or side-veins; maximum two eggs per leaf. Larva (Fig. 69) solitary. Normally entirely parthenogenetic in most of central and northern Europe, where males have so far only been found in the Netherlands (Ad Mol, pers. comm.), but males are apparently more frequent in Greece (Liston 2012, Liston et al. 2015).

##### Distribution.

Widespread in central and southern Europe, from the British Isles, north to Finland (Taeger et al. 2006) and southern Norway (Kiaer 1892); Caucasus (Sundukov 2017); North Africa (Morocco, Middle Atlas: see below).

##### Occurrence in Sweden.

Based on published records: Skåne, Småland (Thomson 1871). Material was examined from Skåne, Halland, Småland, Uppland.

##### Specimens examined.

Bulgaria: 10♀ (SDEI). Germany: 72♀ (SDEI, ZMHB, ZSM). Greece: 4♀ (including DEI-GISHym17935 and 17936), 4♂ (including DEI-GISHym17937) (SDEI). Morocco: Meknes-Tafilalet Region: 1♀, Khénifra 16 km E, 1500 m, 32.93200N, 5.49900W, 18.04.2015, leg. Liston & Prous (SDEI). 3♀, Ifrane 7 km NW, 1590 m, 33.55200N, 5.17500W, 20.04.2015, leg. Liston & Prous (SDEI). Sweden: Skåne: 1♀, Skäralid, 25.05.1965, leg. H. Andersson (MZLU). Halland: 1♀, Kungsbacka kommun, Särö Västerskog, 57.50521N, 11.92572E, 28.04.–14.05.2004, leg. SMTP (NHRS). Småland: 2♀ (NHRS-HEVA000006560 & 6562), no exact locality or date, leg. Boheman (NHRS). Uppland: 1♀ (NHRS-HEVA000003430), Djurgården, 11.05.1937, leg. R. Malaise (NHRS). 1♀, Uppsala kommun, Ekdalens naturreservat, southern hillside, 59.97153N, 18.35495E, 03.–17.05.2004, SMTP (NHRS). 1♀ (NHRS-HEVA000006561), Eknäs, Värmdö, 15.05.1920, leg. Unknown (NHRS).

### *Nematinus* Rohwer, 1911

No reliable key or species treatments are available to date.

### *Nematus* Panzer, 1801

No reliable key or species treatments are available to date.

Prous et al. (2014) radically altered the circumscription of *Nematus*: see also under *Euura*, above. The following synonyms of *Nematus* have been in recent use as valid: *Craesus* Leach, 1817 [= *Croesus*, misspelling], *Hypolaepus* W.F. Kirby, 1882, and *Paranematus* Zinovjev, 1978. Note that most of the species placed in *Hypolaepus* by Lacourt (1999) are now placed in *Euura*.

Currently, fewer than 20 European taxa are considered to be *Nematus* species: *Nematus lucidus*Panzer 1801 (type species), *N. princeps* Zaddach, 1876, *N. umbratus* Thomson, 1871 (=*N. lucens*), all former *Craesus*, and all former *Paranematus*. *Nescianeura noblecourti* Lacourt, 2006 also may belong to *Nematus*.

#### Neodineura

Taxon classificationAnimaliaHymenopteraTenthredinidae

Taeger, 1989

3DF6EB6E80F95666933F4CC46A9C2072

Neodineura Taeger, 1989: 150–151. Type species: Tenthredo (Allantus) arquata Klug, 1816 [= Neodineura arquata], by original designation and the only known species.

##### Description.

Body stocky, similar to *Mesoneura*. Fore wing radial cell divided. Radial cross vein (2r-rs) arises near the apex of stigma and meets the cell 1Rs2; basalis (M) and 1^st^ medial cross vein (1m-cu) strongly converging; M clearly bent only basally; intercostal crossvein (Sc) lying before the junction of M with the Subcosta (Sc+R+Rs); 1^st^ and 2^nd^ medial cross vein (1m-cu and 2m-cu) join the 2^nd^ cubital cell; submedial crossvein (cu-a) meeting medius (Cul) and brachius (lA) almost perpendicularly; anal cell stalked; humeral vein (3A) straight. Hind wing with 2 middle cells, anal cell with long stalk. Inner eye margins slightly converging downwards; distance between the lower eye corners little longer than the maximum eye diameter; clypeus long, shallowly emarginate, in the middle approx. as long as the diameter of a torulus or ca. 1.5 times as long as the distance between the antennal sockets; labrum weakly emarginate on anterior edge; malar space just under half as long as the anterior ocellus; mandibles almost symmetrical, with subapical tooth, in lateral view tapered approximately evenly to the tip. Antenna approx. twice as long as width of head; scape and pedicel distinctly wider than long. Prepectus separated from mesepisternum by a fine line; inner spur of the fore tibia apically divided. Claws bifid, without basal thickening; inner and outer tooth approx. the same thickness, inner tooth slightly shorter.

#### Neodineura arquata

Taxon classificationAnimaliaHymenopteraTenthredinidae

(Klug, 1816)

FC366166C2DD52C4B9FF363E59249433

Tenthredo (Allantus) arquata Klug, 1816: 51. Female (existence of syntypes must be assumed). Type locality: Deutschland. Type specimens lost (Enslin 1914, Taeger 1989). See Taeger (1989) for additional nomenclatural history.

##### Description.

**Figures 119–122.**
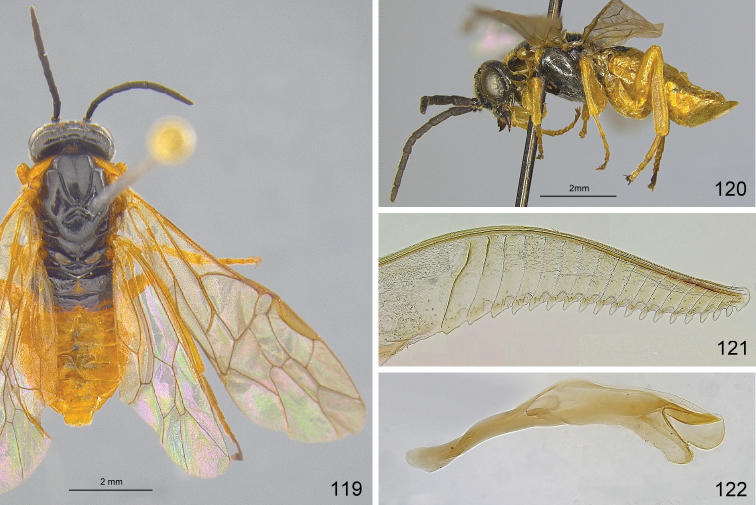
*Neodineura arquata***119** DEI-GISHym15240 ♀ dorsal **120** DEI-GISHym54879 ♂ lateral **121** DEI-GISHym15240 lancet **122** DEI-GISHym54879 penis valve. Scale bar: 2 mm.

This is based on a translation of Taeger (1989), augmented with data gained from examination of specimens which have only recently become available. Body length: female 8.0 mm, male 6.5 mm. Female (Fig. 119) and male (Fig. 120) are similar in colour, apart from the mesopleura: upper mesepisternum pale in female, entirely dark in male. Head and antenna black, except for pale palps and labrum. Thorax dorsally black, with pale tegula and more or less pronotum. Legs entirely pale except more or less for tarsomeres. Wing venation entirely pale brown. Abdomen yellow except more or less for tergum 1. Antennomere 3 little shorter than 4. Postocellar field ca. twice as wide as long; ocellus diameter : POL : OOL = 1 : 1.7 : 2.0; frontal field enclosed by indistinct bulges; supra-antennal groove indistinct; head weakly punctured and shiny; frontal field partly finely wrinkled; thorax slightly more strongly punctured than head. Mesepisternum shiny, with indistinct punctures, evenly covered with rather dense, pale pubescence. Legs relatively thick: femora 3.5 times as long as wide, 0.66 times as long as the tibia; tibia 6.5 times as long as wide and 1.2 times as long as the metatarsus; inner spur of the metatibia nearly as long as the apical width of tibia.

Female: upper half of mesepisternum pale, lower half black. Pronotum, mesepimeron, and metapleura entirely pale. Propleuron edged with black. Head behind eyes subparallel. Antennomere 8 approx. three times as long as wide. Lancet: Fig. 121.

Male: mesepisternum completely black. Pronotum ventrally black. Mesepimeron and metapleura partly pale. Propleuron completely black. Anterior of abdominal tergum 2 also black. Fore wing length 6.5 mm; antennomere 8 3.5 times as long as wide; head behind the eyes clearly narrowed; tergite 8 without special structures; subgenital plate apically rounded. Penis valve: Fig. 122.

##### Similar species.

In the West Palaearctic, *Mesoneura opaca* and *lanigera* are superficially similar in habitus to *Neodineura arquata*.

##### Life history.

Unknown.

##### Distribution.

Only known from Germany, Switzerland (Taeger et al. 2006), the Czech Republic (Beneš and Holuša 2015), and the Russian Caucasus (see below). We are only aware of the existence of four extant collection specimens: three females and one male. Taeger (1989) interpreted the handwritten label data on the only known male (SDEI) as “Sandbg. [Sandberg] 11.V.91”, and thought it likely that the locality was one of several of that name within the then German-speaking territories. Alternatively, it could refer to “Sonderburg” [German name for the Danish island Sønderborg], although the second letter on the label does look more like an “a” than an “o”. Konow received many sawfly specimens, some still in the Konow Collection at the SDEI, from W. Wüstnei, who resided at Sonderburg, and collected from around the late 1880’s to the early 1900’s.

##### Occurrence in Sweden.

No records.

##### Material.

(to the best of our knowledge, the following are the only known extant collection specimens of this species):

Czech Republic [not examined: data from Beneš and Holuša 2015]: Moravia: 1♀, Stolařka Mt., Lhotka, 700 m, 21.05.1998, leg. J. Holuša (NMPC). Germany, or Denmark?: 1♂ (DEI-GISHym54879 / pr.239.(AZ), examined), “Sandbg.” or “Sondbg.”, 11.05.1891 (SDEI). Russia: 1♀ (DEI-GISHym15240, examined), Teberda Reserve, Alibek, 2000 m, 43.32000N, 41.51000E, 22.06.1972, leg. V. Ermolenko (HNHM). Switzerland: 1♀ (DEI-GISHym19777, examined), Solothurn, Rickenbach, 47.34987N, 7.85025E, 560 m, 24.04.1994, leg. Flückiger (SDEI).

#### Nescianeura

Taxon classificationAnimaliaHymenopteraTenthredinidae

Lacourt, 2006

FF0993B09653531E8D6099EDF675E35B

##### Notes.

**Figures 123–127.**
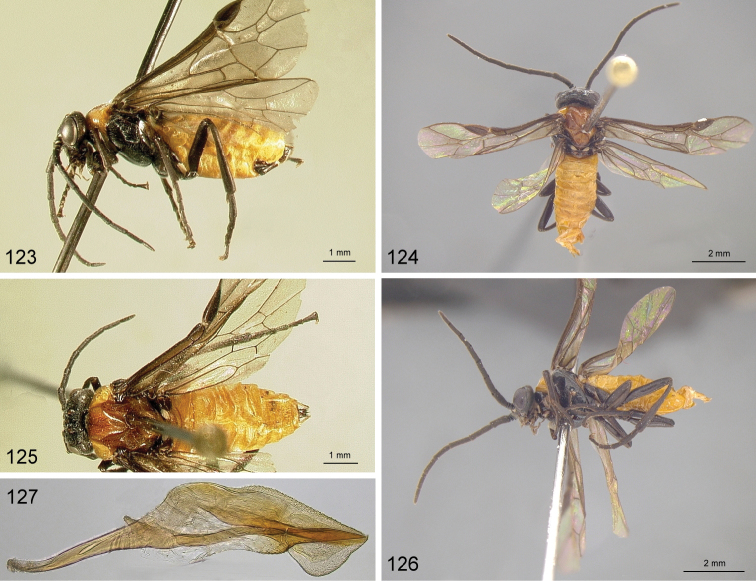
*Nescianeura noblecourti***123, 125** ♀, holotype, France. **124, 126** ♂ DEI-GISHym20933, Germany **127** DEI-GISHym20933 penis valve. Scale bar 1 mm (**123, 125**), 2 mm (**124, 126**).

One species, *Nescianeura noblecourti* Lacourt, 2006, only known from three specimens collected in north-east France and south-west Germany. Females and males, which are similarly coloured, are easily recognised by their distinctive colour pattern (Figs 123–126). Penis valve: Fig. 127. Perhaps a *Euura* or *Nematus* species. See further: Lacourt (2006) and Jansen (2017).

##### Specimens examined.

France: Holotype ♀ (DEI-GISHym20818), Lorraine, Saint-Maurice-sur-Moselle, 26.05.1995, leg. Bernard (MNHN). Germany: 1♀ (DEI-GISHym20932), 1♂ (DEI-GISHym20933), Baden-Württemberg, Grenzach-Wyhlen, Ruschbachtal, 355m, 26.04.–10.05.2008, Malaise trap, leg. Doczkal & Ssymank (SDEI).

#### Platycampus

Taxon classificationAnimaliaHymenopteraTenthredinidae

Schiødte, 1839

F708628863615F97AA1469B2A54A7668

##### Notes.

Two species have been considered to be represented in the West Palaearctic fauna (Taeger et al. 2010): *luridiventris* (see below), and *obscuripes* (Konow, 1896). The latter was described from two females collected in the St Gotthard area, Switzerland. Konow (1896) stated in the original description that *obscuripes* differed from *luridiventris* in its [translated from German] “much smaller head, the apically more weakly emarginate clypeus, and the somewhat shorter third cubital cell, as well as the dark colour of the body and the legs”. Only fragments of one of these specimens now exist. Conde (1937) proposed the synonymy of *obscuripes* with *luridiventris*, basing his concept of *obscuripes* on two female specimens from Piedmont, Italy, leg. Dodero (name of collection not mentioned), and concluded that it is only a dark, alpine form of *luridiventris*. A further female which may belong to *obscuripes*, because it has largely black metafemora, was collected in 1954 in Oberstdorf, Bavaria, by E. Enslin (Manfred Kraus Private Collection). Finally, Weiffenbach (1975) stated that he reared a female *obscuripes* collected on *Alnus viridis*, from Montafon, western Austria, 1800 m. Normally coloured specimens of *luridiventris* are known to occur on *Alnus viridis*, at lower altitudes, in Central Europe (see below). The status of *obscuripes* requires re-assessment, preferably including the use of genetic data.

#### Platycampus luridiventris

Taxon classificationAnimaliaHymenopteraTenthredinidae

(Fallén, 1808)

BB5DC40F0AFB57CDB8FD336A3CA94F6F

Tenthredo alnicola Bechstein & Scharfenberg, 1805: 867. Syntypes, larvae, lost. Type locality: Germany. Synonymy with Leptopus luridiventris by Brischke (1883b: 216). Nomen oblitum after Blank et al. (2009: 47).
Tenthredo luridiventris Fallén, 1808: 115–116. Syntype(s) ♀, not examined (revised by Lindqvist 1956: 9), in MZLU. Type locality: Sweden. Nomen protectum after Blank et al. (2009: 47).
Nematus hypogastricus Hartig, 1837: 184. Syntypes ♀, Deutschland, lectotype ♀ here designated, (GBIF-GISHym3464, images: https://doi.org/10.6084/m9.figshare.4788550), in ZSM. Type locality: Germany. Paralectotype ♀ (GBIF-GISHym3465), in ZSM. Listed in synonymy with Leptopus luridiventris by Thomson (1871: 78).
Nematus alnivorus Hartig, 1840: 27. Syntypes ♀, Norddeutschland, lectotype ♀ here designated (GBIF-GISHym4675) in NFVG. Type locality: Harz, Roßtrappe (Germany). Paralectotype 1♀, in FMNH. Synonymy by Lindqvist (1965: 31–32).
Nematus rufipes Tischbein, 1846: 77. Syntypes ♂♀(?), lost. Type locality: Eutin (Germany). Listed in synonymy with Leptopus luridiventris by Konow (1905: 78).
Leptopus rufipes Förster, 1854: 276–277. Syntypes ♂, Aachen, lectotype ♂ here designated, (GBIF-GISHym3468, images: https://doi.org/10.6084/m9.figshare.4788580), in ZSM. Type locality: Aachen (Germany). Paratype ♂ (GBIF-GISHym3469), in ZSM. Synonymy with Leptopus luridiventris by Brischke (1883b: 216).
Nematus protensus Förster, 1854: 322–323. Syntype(s) ♀, Aachen, lectotype ♀ here designated, (GBIF-GISHym3467, images: https://doi.org/10.6084/m9.figshare.4788595), in ZSM. Type locality: Aachen (Germany).
*Camponiscus Healaei* [sic!] Newman, 1869: 215–217. Syntypes ♂♀, larvae, lost. Type locality: United Kingdom. Synonymy with Tenthredo luridiventris by Cameron (1873: 84).
Nematus Tischbeini [sic!] André, 1880: 120. Replacement name for Nematus rufipes Tischbein, 1846.
Nematus Fennicus [sic!] André, 1880: 133. Syntype(s) ♀, deposition unknown. Type locality: Finland. Synonymy by Forsius (1920: 111).
Nematus alnicola Zaddach in Brischke, 1883b: 188–189. Holotype ♀, “wohl im westlichen Deutschland”, lost. Type locality: Germany(?). Synonymy with Leptopus luridiventris by Brischke (1883b: 216). Secondary homonym of Tenthredo alnicola Bechstein & Scharfenberg, 1805.
Nematus cellularis Brischke, 1884: 138–139. Syntypes ♂♀, Danzig, lost. Type locality: Gdansk (Poland). Primary homonym of Nematus cellularis Dahlbom, 1836. Synonymy with Leptocercus luridiventris by Konow (1901: 89).
Platycampus luridiventris var. pleuritica Enslin, 1915: 322. Syntype(s) ♀, no data, lectotype ♀ here designated (GBIF-GISHym3466, images: https://doi.org/10.6084/m9.figshare.4788727) in ZSM. Type locality: Lisieux (France).

##### Taxonomy.

**Figures 128–133.**
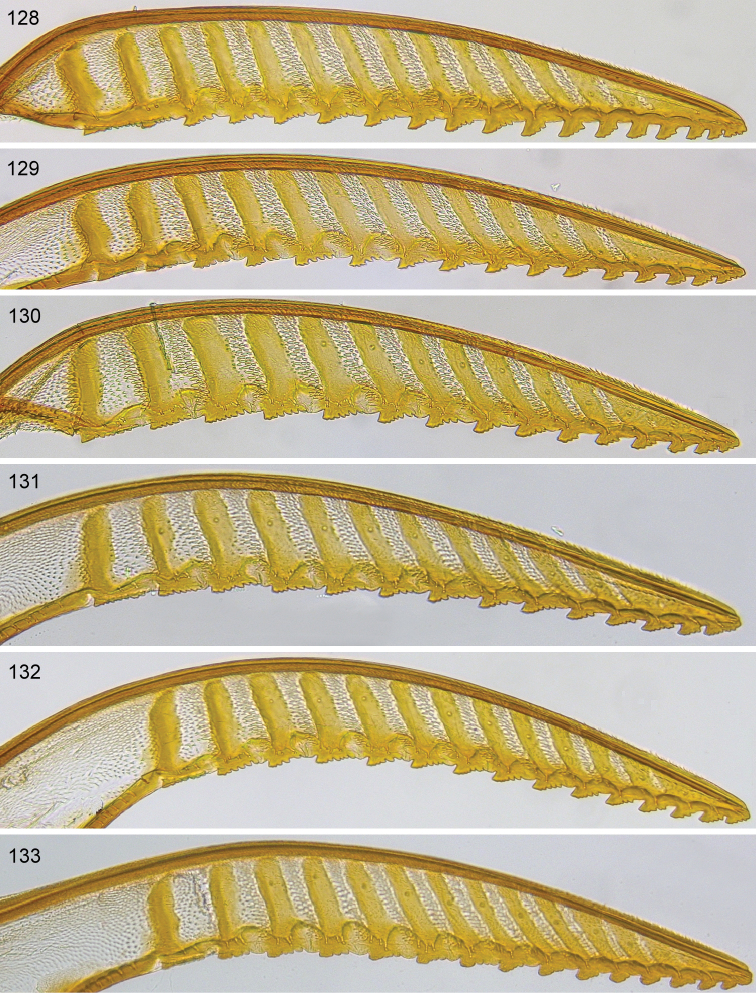
*Playcampus luridiventris*, lancets, variability and wear of teeth **128** DEI-GISHym21133, Sweden, Torne Lappmark **129** DEI-GISHym21134, Sweden, Torne Lappmark **130** DEI-GISHym31937, Sweden, Ångermanland **131** DEI-GISHym31938, Sweden, Småland **132** DEI-GISHym11313, Germany, Mecklenburg-Vorpommern **133** DEI-GISHym31936, Germany, Mecklenburg-Vorpommern, teeth worn.

W. Heitland, H. Pschorn-Walcher and J. Herbst studied European populations of *P. luridiventris* feeding on *Alnus glutinosa*, *incana*, and *viridis*. They found the populations on each host to be genetically segregated (Herbst and Heitland 1994), and that the different hosts correlated with differences in behaviour (Heitland and Pschorn-Walcher 2005), and partly in the morphology of larvae (Heitland and Pschorn-Walcher 1992): setae on the head and body of larvae from *glutinosa* tended to be shorter than of those from *incana*, but setae of larvae from *viridis* usually did not differ from those on *glutinosa*. Our genetic data based on sequences of four genes contradicts, at least partly, the results of Herbst and Heitland (1994). Although six sequenced larvae collected in three different localities (Lower Austria) from three different *Alnus* species do segregate based on mitochondrial COI (1078 bp) into three clusters according to the host plant and locality (maximum distance 2.2%), the nuclear sequences (NaK, POL2, TPI: 5017 bp including introns) are practically identical (only four variable / heterozygous positions, giving a maximal pairwise distance of 0.08%), so that the tree structure for *P. luridiventris* on Fig. 1 is entirely determined by COI. For comparison, nuclear divergence within most other species of Nematinae (based on heterozygous females) is larger, on average 0.2% or up to 1%. In addition, COI sequences of two specimens reared from *A. incana* from Abisko (DEI-GISHym21133, DEI-GISHym21134) are identical to two larvae collected from *A. glutinosa* from Lower Austria (DEI-GISHym21496, DEI-GISHym21497). Since different food plant species can affect gene expression differently in feeding larvae (Yu et al. 2016, Orsucci et al. 2018, Okamura et al. 2019), one can speculate that the allozyme analyses by Herbst and Heitland (1994) were influenced more by differences in the expression of the studied proteins (preferential expression of certain alleles or isoforms) than differences in genetics. Morphologically, we noticed conspicuous differences in the overall shape and spacing of the sawteeth, particularly the apical ones, between the reared Swedish specimens (Figs 128–129) and a German specimen belonging to the other barcoding cluster (Fig. 132). However, examination of further specimens revealed wide variability in the shape and spacing of the sawteeth, with several intermediates (e.g., Figs 130–131), so that finally no clear morphological separation of two groups seemed possible. Perhaps this variability is mainly correlated with geographical occurrence, with a tendency in northern specimens to shorter, more projecting teeth: the lancets of two Abisko specimens (Figs 128–129) have the most clearly projecting and shortest sawteeth (with correspondingly long distances between them), while a specimen from southern Sweden (Småland) has long and flat teeth (more closely spaced) (Fig. 131), and a specimen from Central Sweden is intermediate with regard to the shape of the teeth, although they are widely spaced (Fig. 130). In these examples, the differences are not caused by wear of the saw teeth, because the outlines of the teeth are angular and the denticles are clearly differentiated. A highly worn lancet has rounded edges of the teeth, and the denticles are no longer clearly discernible (Fig. 133). Note that apparent differences in the overall curvature of the illustrated lancets are the result of preparation: each annulus of the lamnium can move slightly, relative to its neighbours, and slight differences in the curvature of the whole lamnium are thus mostly artefacts resulting from preparation. In the light of the foregoing considerations, we conclude that although the three segregates could perhaps be considered to be host plant races [“foodplant races”], as already suggested by Heitland and Pschorn-Walcher (2005), they should certainly not be accorded a formal nomenclatural status.

##### Description.

Body length: female 5.0–7.0 mm, male 4.5–6.0 mm. Female: head black except for palps, and more or less labrum, underside of antennal flagellum, and sometimes more or less scape and pedicel. Thorax black, except for yellow tegula and more or less posteriodorsal edges of pronotum. Sometimes lateral edges of median mesoscutal lobe, and upper mesepisternum pale. Legs pale (orange), with dark metatarsus and apex of metatibia, and more or less dark bases of coxae. Wing venation mostly brown, with centre of fore wing stigma paler. Cerci pale; rest of abdomen from completely black except for obscurely brown area of hypopygium, to all sterna bright yellow, sometimes also with yellow on downturned lateral edges of terga. One reared female from Abisko has dorsal parts of terga 2–4 pale. Variability in the shape of the teeth of the lancet is considerable (Figs 128–133): see also under Taxonomy above. Male: colour similar to female, but pronotum entirely black. Sternum 9 black to pale. Harpes more or less pale.

##### Similar species.

If the nearly complete loop formed by the curved up base of fore wing vein 2A+3A in *Platycampus* is overlooked, then it might be mistaken for *Stauronematus platycerus*, which is similarly coloured and also has bifid claws (but with an additional basal lobe not found in *Platycampus*), or perhaps a *Pristiphora* species.

##### Life history.

Host plants: *Alnus glutinosa*, *incana*, and *viridis* (Heitland and Pschorn-Walcher 1992). Mentions by Lorenz and Kraus (1957) of *Betula*, *Corylus avellana* and *Rubus* as hosts of *luridiventris* are likely to have been based on misidentifications (Zinovjev 1986, Heitland and Pschorn-Walcher 1992). A strictly univoltine species, although some populations exhibit polymodal emergence patterns. Correlated with its highly distinctive larval morphology (Figs 72–73) compared to other nematine genera (Boevé and Angeli 2010), *Platycampus luridiventris* has many peculiar behavioural traits, such as the extremely long time, of approximately three months, taken by the larva to mature (Heitland and Pschorn-Walcher 2005). Oviposition is into the leaf petiole or midrib, with a maximum of three eggs per leaf. The larva is crepuscular according to Heitland and Pschorn-Walcher (2005), and feeds only for very short periods, making holes in the leaf blade, and during the day is normally found immobile on the leaf underside, often in an angle between the midrib and a lateral vein. Sex ratio appears to be normal for netted specimens, i.e., males about as abundant as females, but is heavily skewed towards males in material collected with Malaise traps.

##### Distribution.

Widespread in Europe, from the British Isles to the Balkans, and north to Norway and Finland (Taeger et al. 2006). Earlier published records of *luridiventris* from the East Palaearctic and Oriental Realms, such as by Benson (1963) from Sichuan, China, probably often refer to other species (Zinovjev 1986). For Russia, Sundukov (2017) lists only European areas and the Ural as definite areas of occurrence.

##### Occurrence in Sweden.

Published records: Thomson (1871) wrote “not rare, throughout Sweden”. Material examined from Skåne, Småland, Östergötland, Västergötland, Bohuslän, Södermanland, Uppland, Norrbotten, Torne Lappmark.

##### Specimens examined.

Estonia: 3♀, 1♂ (SDEI, TUZ). Finland: 1♂ (SDEI). France: 1♀, 1♂ (SDEI). Germany: over 100♀ and 150♂ (SDEI, ZMHB, ZSM), including 1♀ (DEI-GISHym11313), Mecklenburg-Vorpommern, Wrangelsburg 16 km SE Greifswald, 54.01611N, 13.59972E, 07.05.2011, leg. H.-J. Jacobs (SDEI); 1♀ (DEI-GISHym31936), Mecklenburg-Vorpommern, Ventschow, 53.78000N, 11.57000E, 09.06.2012, leg. H.-J. Jacobs (SDEI). Poland: 1♀ (SDEI). Sweden: Skåne: 1♂, Simrishamns kommun, Stenshuvuds nationalpark, Stenshuvud-Krivarboden, 55.66035N, 14.27561E, 06–20.08.2004, leg. SMTP (NHRS). 1 specimen, Bökeberg (NHRS). Småland: 1♀ (DEI-GISHym31938), 1♂ (DEI-GISHym31112), Hultsfred, Kloster Gård, 100 m, 57.49700N, 15.87100E, 31.05.2013, leg. Liston, Prous & Taeger (SDEI). 9♀, 2♂, Nybro kommun, Bäckebo, Grytsjöns naturreservat, 56.93148N, 16.08550E, 18.05.–16.06.2006, leg. SMTP (NHRS). 9 specimens (NHRS). Östergötland: 1♂, Ödeshögs kommun, Omberg, Storpissan, 58.33500N, 14.65521E, 28.05–05.07.2005, leg. SMTP (NHRS). Västergotland: 1 specimen (NHRS). 4 specimens (NHRS). Bohuslän: 1 specimen (NHRS). Södermanland: 1 specimen (NHRS). Uppland: 1 specimen (NHRS). Ångermanland: 1♀ (DEI-GISHym31937), Ramvik, 62.87200N, 17.85800E, 04.06.2013, leg. Liston, Prous & Taeger (SDEI). Norrbotten: 1♂ (DEI-GISHym20975), Pajala 8 km NE, 150 m, 67.25200N, 23.54800E, 10.06.2014, leg. E. Heibo (SDEI). Torne Lappmark: 2♀ (DEI-GISHym21133, 21134), Abisko 9 km E (Stordalen), 400 m, 68.35000N, 19.03500E, larvae 26.08.2013, *Alnus incana kolaensis*, emerged 04.2014, leg. Liston (SDEI). Switzerland: 2♂ (SDEI, ZSM). United Kingdom: 1♀ (SDEI).

#### Pristiphora

Taxon classificationAnimaliaHymenopteraTenthredinidae

Latreille, 1810

C06692EC19CB5169A791CE6DEAA72BC5

Pristiphora Latreille, 1810: 294, 435. Type species: Pteronus testaceus Jurine, 1807 [= Pristiphora testacea (Jurine, 1807], by original designation.
Dinematus Lacourt, 2006: 237–238. Type species: Dinematus krausi Lacourt, 2006, by original designation. **Syn. nov.**

##### Notes.

As already suggested by Prous et al. (2017), *Dinematus krausi* probably belongs to the *Pristiphora depressa* species group: see also comments under the species name, below. One of the main reasons for the erection of a genus separate from *Pristiphora* for *krausi*, was the presence of vein 2r-rs in the right fore wing of the holotype (this vein absent in the left wing). The presence of this vein in *Pristiphora* is rather rare but has been observed in at least four other West Palaearctic species: *helvetica* (Benson 1960b), *malaisei*, *robusta*, and *staudingeri* (Prous et al. 2014, 2017). Within *Pristiphora*, these species are only distantly related. In our opinion, no characters exist which will reliably distinguish *Dinematus* from *Pristiphora*, and we therefore propose their synonymy. For further synonymy of genus group names with *Pristiphora* see Taeger et al. (2010) but note that *Stauronematus* is now considered to be a separate genus (Prous et al. 2014). The north-west European species groups and the majority of species of *Pristiphora* were recently revised by Prous et al. (2016, 2017, 2018).

#### Pristiphora krausi

Taxon classificationAnimaliaHymenopteraTenthredinidae

(Lacourt, 2006) new combination

D5986C3C5CBA564E8BCD267266D64CA2

Dinematus krausi Lacourt, 2006: 238–239. Holotype ♀ (MNHN, examined; images: https://doi.org/10.6084/m9.figshare.1157834.v1). Type locality: Saint Maurice-sur-Moselle (Vosges) [France, Lorraine].

##### Notes.

*Pristiphora krausi* is only known from the holotype. Its character combination of bifid claws, in dorsal view short and emarginate valvula 3, and yellow and black colour pattern of head and body, suggest that it may belong to the *Pristiphora depressa* group (Prous et al. 2017). On the other hand, other currently known female specimens of this group have a mostly dark forewing vein C and pterostigma, whereas these are entirely pale in *krausi*. Furthermore, the distal sawteeth of *krausi* are prominently lobed, and markedly flatter in the other species. *Pristiphora ifranensis* Lacourt, 1973, only known from the male holotype (private collection of Thierry Noblecourt, examined), type locality Ifrane (Morocco, Middle Atlas), resembles *krausi* strongly in coloration, including its pale forewing vein C and pterostigma. Based on its penis valve morphology, *ifranensis* has been placed in the *depressa* group (Prous et al. 2017). If further specimens become available for study, the possibility should be borne in mind that *krausi* and *ifranensis* represent the female and male of the same species.

#### Pristiphora malaisei

Taxon classificationAnimaliaHymenopteraTenthredinidae

(Lindqvist, 1952)

78838D4E01C752C48EDE87FB021E7493

##### Notes.

A single larva was obtained in northern Sweden by combing through the leaves of an isolated clump of *Dryas octopetala*, under which an inverted frisbee was held. The plant was growing on an otherwise bare patch of soil at the edge of a road. Gene sequences of the larva are nearly identical to those of *Pristiphora malaisei* imagines collected in the same area. Although the specimen (Fig. 74) is small (approx. total length 3 mm), and has been conserved in 96% ethanol, it seems to resemble the larva of *P. dasiphorae* as described by Zinovjev (1993) much more closely than the larva of *P. malaisei* (see Fig. 86) described in the same paper [under the name *Pristicampus incisus* (Lindqvist), synonymised with *malaisei* by Prous et al. (2017)], in having only three annulets on abdomen segments [six, as described by Zinovjev for *incisa*, on *Potentilla fruticosa*] and very long body setae [much shorter as described by Zinovjev]. Note that *dasiphorae*, so far only associated with *Potentilla fruticosa* as a host and in Europe known only from the Swedish island of Öland, is genetically clearly separable from *malaisei* (Prous et al. 2017). The larva from *Dryas* cannot, therefore, belong to *dasiphorae*. Zinovjev (1993) based his description of the larva of *malaisei* (as *incisus*) on specimens collected in the East Palaearctic (Siberia). Efforts should be made to obtain mature larvae of *malaisei* from northern or subarctic-alpine areas, in order to check the morphology of the larva, and to test the host association with *Dryas*.

##### Specimen examined.

Sweden: Torne Lappmark: 1 larva (DEI-GISHym83704), from *Dryas octopetala*, Abisko National Park (380 m), 68.35300N, 18.76300E, 06.08.2017, leg. Liston & Prous (SDEI).

#### Pseudodineura

Taxon classificationAnimaliaHymenopteraTenthredinidae

Konow, 1885

7FC674D17EA35A1EA28950F14B7DB684

##### Notes.

See Liston et al. (2019b).

### *Stauronematus* Benson, 1953

#### Key to the European species (after Liston, 2007)

**Table:** 

1	**a** Pronotum completely black, or only extreme upper and rear edges brown (Fig. 134); **b** Abdomen entirely black; **c** Mesepisternum more densely pubescent above than below but without extensive entirely glabrous area on lower half (Fig. 134); **d** Hind coxa with at least basal half black (Fig. 134); **e** Wing membrane hyaline; **f** Lancet with ca. 19 teeth (Fig. 136); **g** Penisvalve with ventral margin of paravalva not emarginate (Fig. 138); **h** Body length 5.0–6.5 mm Larval hosts: *Populus* spp., rarely on *Salix*	*Stauronematus platycerus (Hartig, 1840)
—	**aa** Pronotum almost completely pale white or bright yellow, only ventral margins black (Fig. 135); **bb** Abdomen apically more or less pale: in ♀ at least hypopygial area pale brown, sometimes abdomen medially completely pale (yellow); in ♂ subgenital plate and harpes brown; **cc** Mesepisternum with an extensive glabrous area on lower half (Fig. 135); **dd** Hind coxa with only extreme base black (Fig. 135); **ee** Wing membrane slightly infuscate; **ff** Lancet with ca. 16 teeth (Fig. 137); **gg** Penisvalve with ventral margin of paravalva emarginate (Fig. 139); **hh** Body length 5.0–5.5 mm Larval host: *Salix atrocinerea*. *S. purpurea* requires confirmation. Only known from Corsica and Sardinia	***Stauronematus saliciphilus* Liston, 2007**

**Figures 134–139.**
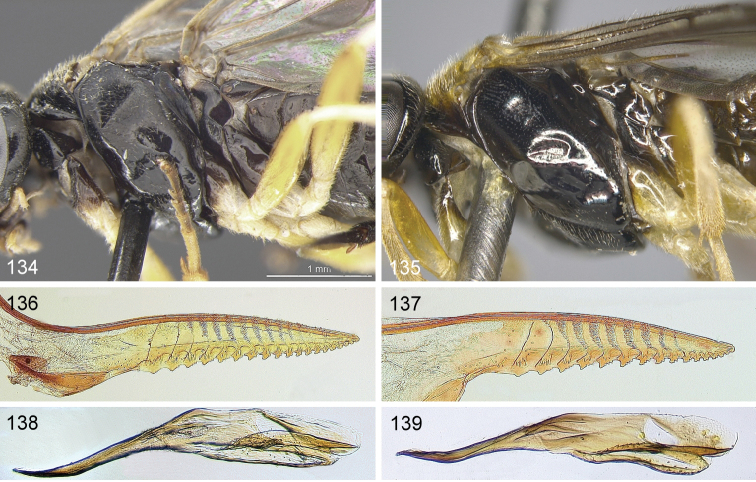
*Stauronematus***134***platycerus* DEI-GISHym19761 ♀ lateral **135***saliciphilus* holotype ♀ DEI-GISHym11427 lateral **136***platycerus* DEI-GISHym11317 lancet **137***saliciphilus* DEI-GISHym11427 lancet **138***platycerus* DEI-GISHym19762 penis valve **139***saliciphilus* DEI-GISHym11435 penis valve. Scale bar: 1 mm (**134**).

#### Stauronematus platycerus

Taxon classificationAnimaliaHymenopteraTenthredinidae

(Hartig, 1840)

222DB83E7FCD5E80AE906E63211DE53C

Nematus platycerus Hartig, 1840: 27. Lectotype ♂, designated by Liston (2007:139), in ZSM (GBIF-GISHym3385, images: https://doi.org/10.6084/m9.figshare.4791952). Type locality: Norddeutschland (Germany).
Nematus vallator Snellen van Vollenhoven, 1858: 191–194, pl. 12. Lectotype ♀, examined, designated by Thomas (1987: 72), in RMNH. Type locality: Leiden (Netherlands). Synonymy with Nematus compressicornis auct. by Cameron (1878: 267).
Nematus cebrionicornis Costa, 1859: 20. Syntype(s) ♂, not examined, most likely in MZFN. Type locality: Camaldoli Hills, near Naples (Italy). Synonymy with Nematus compressicornis auct. by Brischke (1884: 123) (see also Liston 2007: 139).
Nematus callicerus Thomson, 1863: 619–620. Lectotype ♀, designated by Liston (2007:139), in MZLU. Type locality: Ringsjön (Sweden). Synonymy with Nematus compressicornis auct. by Cameron (1885: 55).

##### Description.

Body length: female 4.5–7.5 mm, male 4.5–6.0 mm. Head black, except for mandibles and palpi. Pronotum completely black, or only extreme upper and rear edges brown. Mesepisternum more densely pubescent above than below but usually without entirely glabrous area on lower half. Hind coxa with at least basal half black. Trochanters and femora completely pale (yellowish). Tibia more whitish: pro- and mesotibia and pro- and mesobasitarsus entirely pale, with rest of tarsus darkened. Metatibia with approx. apical third black but spurs pale. Metatarsus black. Wing membrane hyaline; venation largely pale except for dark fore wing stigma. Abdomen entirely black. Female: head in dorsal view subparallel behind eyes. Antennae normal; not laterally compressed. Cerci pale to dark. Lancet: Fig. 136. Male: head in dorsal view behind eyes only slightly contracted. Antennae strongly laterally compressed, flagellomeres ventrally somewhat produced; may be reddish. Penis valve: Fig. 138.

##### Similar species.

When the shape of the claw is overlooked, *Stauronematus* adults are frequently misidentified as *Pristiphora*. The long, thin cerci of female *Stauronematus*, and the shape of the valvula 3 in dorsal view, are however quite different to any West Palaearctic *Pristiphora* species.

##### Life history.

Host plants: mainly *Populus* spp., especially *tremula*, but also *nigra*, *balsamifera*, *deltoides*, *alba*, and many cultivated forms (Pschorn-Walcher and Altenhofer 2000, Brischke 1884, Cavalcaselle 1968); less often on *Salix purpurea* (Pschorn-Walcher and Altenhofer 2000, our own observations). Frequently recorded as bivoltine, but possibly has even three generations in warmer areas. Sex ratio appears to be normal for netted specimens, i.e., males about as abundant as females, but is heavily skewed towards males in material collected with Malaise traps. Oviposition in a double row in the leaf petiole. The larvae eat holes in the leaf blade and surround the feeding site with “palisades” (Fig. 85) made of a dried secretion produced in their mandibular glands.

##### Distribution.

Found through much of continental Europe, from the Iberian Peninsula and Balkans, to Finland and Norway, and also the British mainland (Taeger et al. 2006). According to Sundukov (2017) also occurs in Caucasus, Turkey, Iran, Kyrgyzstan, Kazakhstan, China, Korean Peninsula, and Japan.

##### Occurrence in Sweden.

Published records: Skåne (Thomson 1871), Småland, Uppland, Norrbotten Lule Lappmark (Haris 2009). Material examined from Skåne Uppland.

##### Specimens examined.

France: 2♀ (RMNH). Germany: 23♀ (including DEI-GISHym11317 and 19761), 24♂ (including DEI-GISHym19762) (SDEI, ZSM). Netherlands: 4♀, 6♂ (RMNH). Portugal: Aveiro: 1♀, Castelo de Paiva 7 km SSW, 260 m, 41.00033N, 8.27777W, 14.05.2012, leg. Blank, Jacobs, Liston & Taeger (SDEI). Spain: 1♀, 1♂ (SDEI). Sweden: Skåne: 1♂, Malmö, Limhamns Kalkbrott, 55.56760N, 12.93283E, 9.06–25.10.2007, leg. B. W. Svensson & Co. (MZLU). 1♂, Malmö, Limhamns Kalkbrott, 55.56760N, 12.93283E, 27.07.–16.08.2009, leg. B. W. Svensson & Co. (MZLU). Uppland: 1♂, Haninge kommun, Tyresta, Urskogsslingan, hällmark, 59.17685N, 18.24690E, 04–26.08.2004, leg. SMTP (NHRS). 1♂, Huddinge kommun, Sofielunds återvinningsanläggning, avlastningsstation, 59.17656N, 17.99379E, 18.05.–07.06.2004, leg. SMTP (NHRS). 1♂, Älvkarleby kommun, Marma skjutfält, east of Sköldvägen/Kanonvägen, 60.52431N, 17.45151E, 17.06–02.07.2003, leg. SMTP (NHRS). 1♀, 1♂, Älvkarleby kommun, Båtfors, between Milsten and Båtforstorpet, 60.46077N, 17.31782E, 17.06.–03.07.2003, leg. SMTP (NHRS). 1♂, same locality as previous, 14.06.–04.07.2005, leg. SMTP (NHRS). 4♂, Uppsala kommun, Ekdalens naturreservat, southern hillside, 59.97153N, 18.35495E, 07–21.07.2003, leg. SMTP (NHRS). 1♂, same locality as previous, 04–18.08.2003, leg. SMTP (NHRS). 2♂, same locality as previous, 18.08.–01.09.2003, leg. SMTP (NHRS). 1♂, same locality as previous, 02.–16.06.2004, leg. SMTP (NHRS).

## Discussion

The conclusions on the phylogeny of Nematinae reached by Niu et al. (2019), based mainly on morphological characters, differ substantially from our results, which are based on molecular data. In our opinion the methodology and data analysis on which their results are based are both seriously flawed. Their results are also affected by misinterpretations of previously published work by other researchers, particularly the papers by Nyman et al. (2006) and Prous et al. (2014). Niu et al. (2019) failed to mention that many of the deepest splits within Nematinae were poorly supported (low statistical support and conflicting relationships in different analyses), although this was acknowledged by both Nyman et al. (2006) and Prous et al. (2014). At the same time, monophyly of Nematinae (including “Hoplocampinae”) was strongly supported in all analyses. In the absence of clear evidence to the contrary, there is no justification for the proposal of alternative classifications: Niu et al. (2019) have not provided such evidence, because they rely solely on the classification proposed by Wei and Nie (1998). Wei and Nie (1998) claimed that their “cladistic analysis” of “Tenthredinoidea” (i.e., Tenthredinidae as currently understood) was based on a “…huge data matrix”, but that “…the complicated analysis process are omitted here for limited space and they will be reported in detail in a separated monograph.” We are unaware of any sources or publications which provide these data. Wei and Nie (1998) basically elevated many existing taxa to higher rank (tribes to subfamilies, subfamilies to families etc.) with little or no increase in information content. In the absence of publicly available evidence, we are sceptical that Wei and Nie (1998) managed to create a highly informative morphological data matrix that could be used to propose a well-supported and stable phylogeny of Tenthredinidae. The cladistic analyses by Vilhelmsen (2015), based on 146 morphological characters, demonstrate how difficult it is using such methods to achieve a high level of statistical support and stability for phylogenies within Tenthredinidae. At the same time, the statement by Niu et al. (2019: page [2]) that the results of Prous et al. (2014) were based “only on 400-bp sequences of the barcode region”, is simply wrong. As clearly described in Prous et al. (2014: 3) there were two datasets based on four genes (two mitochondrial and two nuclear), one of them (134 specimens) with little missing data (19 specimens missing one gene and seven specimens missing two genes) and the second one (79 specimens) with more missing data (21 specimens missing one gene, eight specimens missing two genes, and 15 specimens missing three genes). This approach was adopted so that type species of some genera for which only one gene was available could be included in the analyses (only one specimen in the second dataset had 422 bp of COI, all others had at least 658 bp of COI). In the end, the new data presented by Niu et al. (2019) are irrelevant to their discussion on the classification of the Nematinae, because of completely inadequate taxon sampling: they analysed only two specimens of Nematinae. Their data are in fact consistent with all previously proposed classifications, not just with Wei and Nie (1998) as they stated.

Although the Nematini and Dineurini both comprise a relatively large number of genera, the large majority of Holarctic nematine species belong to just two genera of Nematini, *Euura* and *Pristiphora*. The proportional representation of genera and species in the Oriental Realm is at present unclear, but compared to the Holarctic Realm, existing data point to a lesser number of *Euura* species, and more *Pristiphora*, while the number of species belonging to diverse genera of non-Nematini may also be greater (Taeger et al. 2010). At the same time, although the number of still undescribed nematine species inhabiting the mountains of the Oriental Region can only be guessed at, it seems unlikely that Nematinae make up such a high proportion of the Oriental sawfly fauna as of the fauna of northern regions of the Holarctic. Outside the Holarctic and Oriental Realms, the Nematinae is represented naturally only in the northern regions of the Neotropical Realm, by a few species of *Pristiphora* (Taeger et al. 2010).

As noted above, the striking abundance and species diversity of nematine sawflies in the northern parts of the Palaearctic, including Fennoscandia, results mainly from the presence of numerous species of *Euura* and *Pristiphora*. Although several factors probably contribute to this pattern (Bogacheva 1994, Kouki et al. 1994), it has long been apparent that at progressively high latitudes in the northern hemisphere *Salix* species are of increasing importance over other plant taxa as hosts of sawflies, particularly Nematinae (Malaise 1931b). On the other hand, it is important to remember that many other plant taxa are hosts of sawfly larvae in the north. An example is our indication that *Dryas octopetala* is a host plant of *Pristiphora malaisei* in the more northern and upland parts of the range of this sawfly species. Currently, this is only the second sawfly species to have been found on this host, the other being the allantine *Empria alpina* Benson (Prous et al. 2011). However, based partly on our own experiences during field-work, we suspect that the relative difficulty of collecting larvae from low-growing potential hosts such as *Dryas*, other herbaceous Rosaceae, Polygonaceae, Fabaceae, grasses and sedges, etc. as opposed to shrubby *Salix*, may have led to at least a slight underestimation of the significance of the former as host plants in the northern nematine fauna. Furthermore, although *Betula* species are clearly the second most frequently used hosts of Nematinae in northern Fennoscandia, most published observations and data are for the tree-birch Betula pubescens var. pumila (e.g., Tenow 1963), whereas surprisingly little has been published about the sawfly fauna of *Betula nana*.

As can be seen from the key to larvae, the larvae of Nematinae exhibit a high level of morphological variability. This is expressed, for example, in the number of dorsal annulets of abdomen segments varying between three and six. By contrast, all European Tenthredininae larvae have seven annulets, six in Selandriinae [only *Dolerus*] or seven, six in each Athaliinae and Allantinae (Lorenz and Kraus 1957). Only among the Blennocampinae is this character similar in variability to the Nematinae: Blennocampinae have 4–6 annulets, excluding the leaf-mining taxa, in which the number is reduced to two. The variability in Nematinae is all the more remarkable because conspicuous differences such as the number of annulets apparently occur even between species which are certainly quite closely related, such as within the *Pristiphora malaisei* species group. In the Blennocampinae, differences in the number of annulets are usually regarded as generic characters (Lorenz and Kraus 1957).

Although the genera which we have treated in this paper are comparatively species-poor, cases nevertheless occur of the sort of taxonomic problems which are regularly encountered in the much larger genera *Pristiphora* and *Euura*. An interesting example is *Platycampus luridiventris*, where three different (mitochondrial) genetic lineages exist. Earlier studies on this species concluded that genetic segregation was correlated with differences in host plant use, behaviour, and partly even the length of setae of larvae. Our own genetic data partly conflicts with this conclusion. Perhaps the apparent differences are caused by differential gene expression: a sort of host plant conditioning. At present, there are no compelling reasons to treat the lineages as separate taxonomic entities. A similar situation may occur in several groups of closely related nominal species of *Euura*, such as the gall-makers of the *dolichura* group and *oblita* group (*ischnocera* complex), which are thought to be highly host specific, but often exhibit neither clear morphological nor genetic differences (Liston et al. 2017).

## Supplementary Material

XML Treatment for Hemichroa australis

XML Treatment for Hemichroa crocea

XML Treatment for Mesoneura opaca

XML Treatment for Neodineura

XML Treatment for Neodineura arquata

XML Treatment for Nescianeura

XML Treatment for Platycampus

XML Treatment for Platycampus luridiventris

XML Treatment for Pristiphora

XML Treatment for Pristiphora krausi

XML Treatment for Pristiphora malaisei

XML Treatment for Pseudodineura

XML Treatment for Stauronematus platycerus
